# Using Machine Learning Technology (Early Artificial Intelligence–Supported Response With Social Listening Platform) to Enhance Digital Social Understanding for the COVID-19 Infodemic: Development and Implementation Study

**DOI:** 10.2196/47317

**Published:** 2023-08-21

**Authors:** Becky K White, Arnault Gombert, Tim Nguyen, Brian Yau, Atsuyoshi Ishizumi, Laura Kirchner, Alicia León, Harry Wilson, Giovanna Jaramillo-Gutierrez, Jesus Cerquides, Marcelo D’Agostino, Cristiana Salvi, Ravi Shankar Sreenath, Kimberly Rambaud, Dalia Samhouri, Sylvie Briand, Tina D Purnat

**Affiliations:** 1 Department of Epidemic and Pandemic Preparedness and Prevention World Health Organization Geneva Switzerland; 2 Citibeats Barcelona Spain; 3 Artificial Intelligence Research Institute Spanish Council for Scientific Research Cerdanyola Spain; 4 Information Systems for Health Evidence and Intelligence for Action in Health Pan American Health Organization and World Health Organization Regional Office for the Americas Washington DC, DC United States; 5 Risk Communication and Community Engagement Unit Health Emergencies Division World Health Organization Regional Office for Europe Copenhagen Denmark; 6 Country Health Emergency Preparedness and International Health Regulations (2005) World Health Organization Regional Office for Eastern Mediterranean Cairo Egypt

**Keywords:** infodemic, sentiment, narrative analysis, social listening, natural language processing, social media, public health, pandemic preparedness, pandemic response, artificial intelligence, AI text analytics, COVID-19, information voids, machine learning

## Abstract

**Background:**

Amid the COVID-19 pandemic, there has been a need for rapid social understanding to inform infodemic management and response. Although social media analysis platforms have traditionally been designed for commercial brands for marketing and sales purposes, they have been underused and adapted for a comprehensive understanding of social dynamics in areas such as public health. Traditional systems have challenges for public health use, and new tools and innovative methods are required. The World Health Organization Early Artificial Intelligence–Supported Response with Social Listening (EARS) platform was developed to overcome some of these challenges.

**Objective:**

This paper describes the development of the EARS platform, including data sourcing, development, and validation of a machine learning categorization approach, as well as the results from the pilot study.

**Methods:**

Data for EARS are collected daily from web-based conversations in publicly available sources in 9 languages. Public health and social media experts developed a taxonomy to categorize COVID-19 narratives into 5 relevant main categories and 41 subcategories. We developed a semisupervised machine learning algorithm to categorize social media posts into categories and various filters. To validate the results obtained by the machine learning–based approach, we compared it to a search-filter approach, applying Boolean queries with the same amount of information and measured the recall and precision. Hotelling *T*^2^ was used to determine the effect of the classification method on the combined variables.

**Results:**

The EARS platform was developed, validated, and applied to characterize conversations regarding COVID-19 since December 2020. A total of 215,469,045 social posts were collected for processing from December 2020 to February 2022. The machine learning algorithm outperformed the Boolean search filters method for precision and recall in both English and Spanish languages (*P*<.001). Demographic and other filters provided useful insights on data, and the gender split of users in the platform was largely consistent with population-level data on social media use.

**Conclusions:**

The EARS platform was developed to address the changing needs of public health analysts during the COVID-19 pandemic. The application of public health taxonomy and artificial intelligence technology to a user-friendly social listening platform, accessible directly by analysts, is a significant step in better enabling understanding of global narratives. The platform was designed for scalability; iterations and new countries and languages have been added. This research has shown that a machine learning approach is more accurate than using only keywords and has the benefit of categorizing and understanding large amounts of digital social data during an infodemic. Further technical developments are needed and planned for continuous improvements, to meet the challenges in the generation of infodemic insights from social media for infodemic managers and public health professionals.

## Introduction

### Background

From the outset of the COVID-19 pandemic, the infodemic, the excess of information, including misinformation and disinformation that can result in confusion or impact on health attitudes and behaviors during health emergencies, has been of keen interest to those involved in emergency response [1]. The infodemic can have a poor impact on public health outcomes [2,3], and there is evidence that those who are most at risk may be most vulnerable to infodemic [4]. During the pandemic, social and other digital media have allowed for the rapid dissemination of an overwhelming amount of information that can elongate or amplify outbreaks and reduce the effectiveness of epidemic response efforts and interventions [1]. People may feel confused by who to trust; may be confronted by outdated or incorrect information; or may be exposed to, share, or act on misinformation and disinformation. Having a comprehensive infodemic management strategy that includes integrated infodemic insights generation from offline and web-based social listening sources, as well as data sources in the health information systems and outside the health sector, may help build trust in governments and health authorities and help people understand and accept the pandemic response [5]. The World Health Organization (WHO) public health research agenda for infodemiology outlines priority research questions structured under 5 key thematic areas [6]. These themes have recommendations for preparedness and monitoring of the infodemic and for detecting and understanding the spread of infodemics. Better social media analysis tools and metrics are needed to support infodemic managers to understand and respond to an infodemic during a health emergency.

### Social Media Analysis for Public Health

Social listening is often understood as an approach from marketing and communication to glean insights from analysis of social media channels [7], whereas in public health, social listening is often more broadly, including using data sources from the health system, sociobehavioral studies, community feedback, as well as nontraditional sources such as mobility data [6,8]. Social listening for public health involves infodemic insights generation, which is analyzed in an integrated manner across data sources from social listening, health information systems, and outside the health system [9]. Social listening and infodemic insight generation include the analysis of social media, traditional media, and other data sources—such as user search trends, epidemiological data, community feedback, and sociobehavioral data—to identify, categorize, and understand perceptions, questions, concerns, information voids, and narratives expressed and circulating in communities [9].

Social listening, integrated analysis of infodemic intelligence, and generation of infodemic insights and infodemic management recommendations are the first steps in providing evidence to manage the infodemic [10]. When analyzing social media, understanding the source, velocity, and volume of global social media information trends can help inform prebunking and debunking initiatives, fill information voids, develop user experience and digital resilience strategies, and inform infodemic responses [5,9]. Misinformation shared on social media can quickly cross international borders and platforms, with the same claim presented in different ways and contexts to users on YouTube or Facebook for example [11].

The WHO has previously reported on using artificial intelligence (AI)–driven social listening to deliver actionable infodemic insights [9] and on the development and validation of a public health social listening taxonomy [12]. The COVID-19 public health taxonomy was designed to provide a practical and structured approach to identifying narratives shared on digital media [13], and taxonomy-driven data analysis and integration have since been applied to other outbreaks, such as mpox [14]. Developed by public health and digital health experts, the taxonomy enables data to be filtered into categories, allowing for the identification of where the global conversation is growing and what the information voids or issues of concern may be.

Faced with millions of data points, AI can help filter data into these specific categories, as well as filter within the categories by questions or demographic identifiers. This enables an analyst to quickly see the signal through noise and obtain meaningful insights. Although there is significant potential in pandemic response, there have been calls for the application of AI to be grounded in ethical and multidisciplinary practice [15]. There is a need for greater cooperation between domain experts and AI practitioners, that tools be adapted from existing tools and aim to reduce, rather than add to, the workload of health care workers; that systems be adapted to the needs of low- and middle-income countries; and that global solutions with local adaptability options are developed [15]. In addition, there is a need for any application of AI to undergo ethical assessment, including users’ rights to privacy, protecting journalistic sources, and preventing mass surveillance.

### Challenges With Existing Social Media Analysis Systems

Although the need for social understanding amidst the COVID-19 pandemic and infodemic is paramount, traditionally, social media monitoring systems have been established for commercial purposes and brand management rather than providing insights that can inform public health action [16]. There are inherent challenges for public health analysts attempting to navigate these systems to quickly find actionable insights to guide their work. Systems can be costly, which may be a barrier, particularly to people working in lower-resources settings, and may include limited language coverage, data sources, data representatively, privacy, geographical bias, and prioritize “noise” over “signal.” Here, we briefly outline these challenges.

Traditionally, social media monitoring tools have the strongest analytical capability in English. In some cases, this is followed by other widely spoken languages, and in many cases, no coverage of the long tail of minority languages, or adaptation to dialects or sociolects or local references. This can be problematic for analysts seeking to understand global narratives. Even if an additional language is included in the analysis tool, there may be many differences in the local references, which may not be captured by pretrained models or predefined dictionaries. This problem is further exacerbated in a fast-changing conversation topic, such as COVID-19, because vocabulary and new terms are constantly entering the conversation in each language as the situation evolves. Although some dedicated research on the COVID-19 infodemic has been conducted on a local level in India [17], Croatia [18], and Malaysia [19], to our knowledge, there have been few broader solutions covering multiple languages.

The diversity of data sources can be a challenge with some platforms prioritizing certain social media platforms, and principally, Twitter, because of data availability, which may have limitations in the representativity of the population [2] as well as experience changes in the quality and content on the platform due to changes in content moderation policies and platform user experience. Alternative text sources, such as surveys, SMS text message responses, call transcripts, and chatbot questions, often cannot be analyzed by commercial systems, meaning valuable data may be missing from the analysis data set. Maintaining user privacy is an important ethical consideration in public health. Within a branding context, identifying individuals to target with messages or advertisements can be a priority; however, identifying individual users may go against privacy protection principles in the public health context [15,20].

Understanding this representatively is important for ensuring community coverage and developing appropriate actions. A common issue is that in many countries, women’s voices are underrepresented in the overall sample. The data results from social media analysis may indicate that a given topic is important, but this may be skewed toward perspectives from men [21]. In addition, being able to differentiate data based on country level can help reduce geographical bias, as data can be skewed toward USA-centric data, resulting in an underrepresentation of other countries.

### Analytic Approaches to Categorization

A key challenge for public health analysts is how to quickly filter through the “noise”—conversation volume, interactions volume, mentions volume, top influencers, to find the “signal”—the actionable insight that can be identified for the purposes of infodemic management [12]. Finding these signals requires the analyst to look beyond the emphasis on high-engagement posts, rising narrative detection, common questions, sex gaps, and trust indicators. Filtering these narratives through a public health taxonomy enables data to be grouped into relevant categories, enabling analysts to quickly see where the conversation is growing and the directionality.

There are various ways to accomplish this, such as the use of Boolean queries with keywords or natural language processing (NLP). The emergence of NLP models, such as bidirectional encoder representations from transformers (BERT) models [22], and the democratization of those models with open-source hubs, such as the HuggingFace platform [23], enables scientists to implement automatic categorization methods based on machine learning algorithms and deep learning methods. One machine learning approach is supervised learning, which refers to training algorithms from already labeled data. Whereas unsupervised learning relates to classification without any human input, the semisupervised learning paradigm combines a small amount of labeled data provided by a human expert with a large amount of unlabeled data.

Although other studies have reported the use of NLP and automatic categorization for specific components of COVID-19 data [24,25], this is the first study to apply semisupervised machine learning to all COVID-19 narratives. This paper describes the development of the Early AI-Supported Response with Social Listening (EARS) platform [26], data sourcing and collection from December 2020 to February 2022, and how AI was used to filter and categorize data to inform infodemic insights.

## Methods

### The WHO EARS Platform

The WHO EARS platform was developed and piloted to allow infodemic managers to access real-time information on how people are talking about COVID-19 on the web. The data are collected from multiple sources and combined into a user-friendly platform. Data are categorized as a public health taxonomy developed by experts to enable social understanding of COVID-19 pandemic narratives on social media platforms. This taxonomy was informed by previous work [12,13] but developed bespoke for this project and continually reviewed and revised. The taxonomy has 5 main global topics and 41 different categories. The categories are regularly reviewed and revised. The 5 main topics related to the cause of the virus, illness, treatment, interventions (including prevention), and information and misinformation. Multimedia Appendix 1 lists all categories and definitions.

Data visualizations on the platform include combinations of countries, categories of conversation, questions, and gender segments. Data can be shown by which narratives are most prominent, rising in prominence, or outliers compared with other countries in the platform. The public dashboard and public application programming interface are available for aggregated and anonymized data by all users [27], while a private dashboard is being piloted internally by WHO staff and selected partners, with anonymized and granular-level data. The dashboards are updated daily with new data and are intended to assist public health professionals in understanding the narratives and needs of the public to inform policy, communications decisions, or emergency response recommendations. The project was validated and piloted in 30 countries (Multimedia Appendix 2) and 9 languages (English, Spanish, French, Portuguese, German, Italian, Bahasa Indonesian, Thai, and Arabic). For the purposes of setting up and validating the pilot first version of the platform, languages and countries were selected after an internal review of countries that lack access to high-quality language-specific analysis of languages to COVID-19, which at the same time had sufficient data volume to enable analysis by the EARS system.

### Data Sources and Data Collection

The platform collects daily data from web-based conversations in publicly available sources, including Twitter, Facebook Public Pages, and other common web content. However, there are differences in how people use these platforms. Twitter users often share information in real time and react to current events, trends, or topics that are popular in the moment, so information spreads quickly, allowing some events to be detected before they may appear in news outlets. Although Facebook can also be a place for spontaneous discussions, comments tend to be more associated with recent news events or articles, or institutional campaigns. Owing to the country-level analysis, data were collected only where geo-located metadata were available. Examples of common web sources include web-based forums, news comments, blogs, as well as sources such as Reddit, YouTube, and 4Chan. In addition, there are >1000 additional sources of public web- and interest-based communities whose volume varies per country or may be country specific, such as Mumsnet in the United Kingdom, Buriramexpat in Thailand, or Mutdawl in Egypt. These data are collected through the Citibeats platform via their partnerships with data providers, and all analyses focus on aggregated results instead of individual users. A query was developed to capture the COVID-19 data for each country and language. Multimedia Appendix 3 includes the search terms used in each country.

Although Twitter provides most of the data on the platform, efforts have been made to diversify the data collection as much as possible and to apply different methods, such as gender disaggregation, to mitigate any potential biases. To reduce the impact of viral events on Twitter, retweets are not collected, and the aim is to give the same weight and importance to any opinion published on Twitter without granting a more significant position to the most influential and viral voices.

### Taxonomy Classification

#### Overview

After data are collected, they are categorized to the taxonomy using a machine learning algorithm. The main approach to classifying posts relies on a semisupervised learning method that relies on a measure propagation algorithm [28]. Semisupervised learning is a machine learning approach that combines labeled and unlabeled data to train models. It uses additional unlabeled data to improve generalization and leverage the underlying structure of the data. By incorporating unlabeled data, semisupervised learning algorithms can achieve better performance and scalability compared with using only labeled data [29]. This is useful when the labeled data are limited or expensive to obtain.

Measure propagation [28] is a more advanced version of the well-known label propagation algorithm [30]. Label propagation starts with a set of points and a graph structure connecting those points. This algorithm assigns labels to unlabeled data points by propagating the labels in the graph. After the propagation is completed, every data point is assigned to a specific label. Measure propagation is the probabilistic version of label propagation. Instead of propagating labels, measure propagation assigns to each data point a probability distribution over its labels based on the labels of the neighboring nodes in the graph.

We feed the algorithm with texts collected from social media platforms and carefully chosen sets of keywords for each category (eg, a category of fruit would have apple, banana, or grapes as keywords). A keyword can be 1 word, such as “symptom” or several words, such as “patient zero.” Once the keywords were defined for the English language, they were translated and adapted to equivalent expressions to the other 8 languages. A list of keywords for each category in Spanish and English is provided in Multimedia Appendix 4.

In training the algorithm, a weighted graph is built from the texts based on the co-occurrences between words. The nodes of the graph are the texts from social posts, and the lines between the nodes represent the similarities between those social posts computed from the co-occurrences of words used between those social posts. Next, the algorithm uses a propagation method across the graph nodes to label the texts that are most likely to belong to this category. In this case, a sentence mentioning 3 fruits would have a high probability to fall into the fruit category. Then, to determine if other texts fall into the category, the algorithm propagates across the graph nodes, updating the categorization by considering the texts it has labeled in the previous step. The algorithm returns to the third step until the classification of the graph nodes converges to a stable state. The propagation step occurs daily.

Where a post does not fall into one of the categories, it is considered as “noise” and excluded from the data set. This occurs when the classification algorithm finds no relationship with any of the categories. Examples of posts considered as noise are posts containing only hashtags or user mentions or posts that contain a word from the taxonomy but are not related to the subject of the study.

#### Testing the Algorithm

To assess the performance of the algorithm in classifying data into categories, we computed the precision, recall, and *F*_1_ metrics. Recall represents the amount of relevant information retrieved from social media posts, whereas precision refers to how much of the retrieved information is relevant [31]. The *F*_1_-score is the harmonic mean between recall and precision. This metric displays the effectiveness of the retrieval model and considers recall and precision of equal importance and is widely used in machine learning [32-34].

To compare this method with related works [13], we created a baseline based on Boolean queries. We fed the Boolean queries and the machine learning algorithm with exactly the same keywords related to each category and computed the same performance metrics. To calculate the metrics for both approaches, we randomly sampled the data collected from the Citibeats platform and annotated them according to the taxonomy categories. The data were sampled between January and February 2022. For validation, we limited the data to Spanish (from Colombia, Spain, and Mexico) and English (from Kenya, India, and the United Kingdom) languages. For each language, the reviewers manually labeled the data, with a single reviewer leading each language. A second reviewer reviewed a random sample (15% of each language data set) to ensure the quality of annotations; any disagreements were resolved via discussion, and the annotations were rereviewed. The reviewers labeled each text according to the categories they should fall into. For each category, we considered the following text:

1. True positive (TP)—text correctly classified
2. False positive (FP)—text did not belong in the category it was assigned.
3. False negative—text belongs to the category but was not classified into it.

These data were then used to calculate the precision = TP / (TP + FP); recall = TP / (TP + false negative); and finally, the *F*_1_-score = (2 × precision × recall) / (precision + recall). As there were 41 taxonomy categories, we computed this for each category and as an overall average of the scores across all categories. Hotelling *T*^2^ [35] was used to determine whether the difference between the categorization methods (Boolean or algorithm) was statistically significant. As Hotelling *T*^2^ is an *omnibus* test, it indicates whether the combined dependent variables are statistically significantly different in terms of the 2 classification methods. A significance level of *P*<.05 was used.

### Demographics and Intents

To enable a more fine-grained analysis and understanding of the representativity of the data, we developed a tool to filter by post origin, poster type (individual or institution), and gender. The ability to detect which posts are from people and which posts are from institutions (including official accounts) is important as institutions’ discussions bring relevant information about COVID-19’s narratives but may differ from citizens’ discussions.

The gender classifier is inferred from the web-based data using a deep learning method. The system uses indicators, such as the author’s name and biodescription, and makes a final determination of gender probability. Women are underrepresented in the media [36], and the ability to filter by gender can help isolate and amplify the voices of women.

The tool for gender differentiation extrapolated on existing methods [21,32]. A limitation of the platform is that it currently only supports dominations of men or women, and including nonbinary categories is planned for future integration. For this research, we calculated the known gender proportion of users in our data set and compared it with country data from the study by Hootsuite [37,38] to determine the proportionality of users in our database to the population.

To extract more insightful and actionable information from the analyses, we also implemented a query detector to detect whether citizens’ texts contained questions. The detector is a multilingual machine learning algorithm based on multilingual BERT architecture [39] that detects whether social posts carry a question. The classifier discards from the classification all rhetorical questions, quotations, advertisements, newspaper headlines, or questions with an answer. We used Mexico and the United Kingdom as comparator examples for reporting on all demographic filters.

### Category Analysis

Calculating the velocity of data change is important for identifying information voids and for the early identification of changes in narratives. Velocity refers to the percentage increase in narratives in a certain category. To identify the velocity change in a category, we compared the weekly volume of social media posts with the moving average volume over the last 4 weeks. Intuitively, the moving average series is smoother, less subject to variation, and represents the trend of volume over time. It also limits FP velocity alerts when we have an alternate series. A category is flagged as a velocity alert when the new weekly volume change is a minimum of 15% higher than the mean of the previous week. A 15% threshold was established based on analysts’ experience with velocity data. The formula used to calculate the weekly velocity rates is as follows:



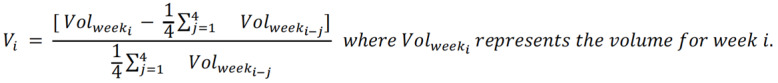



Each week, a velocity (*V*) is computed according to current and previous weekly data volumes (*Vol*). The velocity for the week *i* (*V_i_*) is the relative difference between the current week data volume (*Vol_i_*) and the mean of the data volume across the last 4 weeks.

The 4-week period was chosen because it provided enough time to have a stable comparison while still maintaining recency. The specific number of weeks was defined after testing with higher and lower numbers of weeks and reviewing the relevant changes. After applying data analysis to the different trials per week, the best results were obtained with the 4-week comparison, providing alerts most aligned with the insights needed to inform decision-making regarding COVID-19.

**Figure 1:**
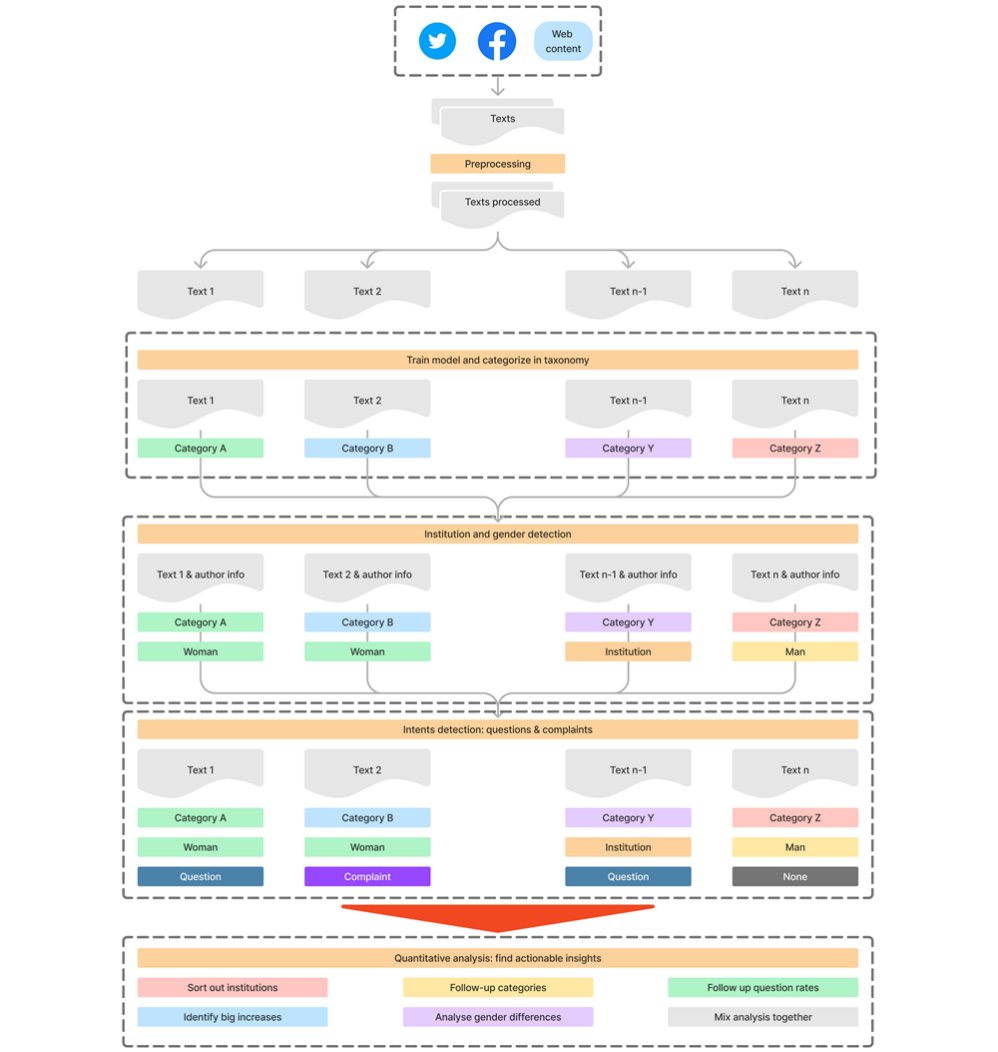
Early Artificial Intelligence–Supported Response With Social Listening (EARS) data processing and analysis method. Texts are first collected from social networks, we then train the semisupervised algorithm to categorize the texts to the taxonomy. Following this, the organization and gender and question intentions are applied, and finally, the data generated is ready for user analysis.

Velocity metrics can be combined with other filters to analyze category velocity through demographic segments, question rates, or combining demographic segments with question rates. We present the results of the velocity data by filtering for 2021 for Mexico and the United Kingdom. Figure 1 summarizes the entire data processing and analysis pathway, including data collection, categorization into the taxonomy, demographics segmentation, intents detection, and category analysis.

## Results

### Data Collection

Data were collected for English-, French-, Portuguese-, and Spanish-speaking countries from December 2020 to February 2022. Arabic, Bahasa Indonesian, German, Italian, and Thai languages were added in September 2021; thus, only data from this time point onward are available, resulting in a lower data volume in comparison. In total, 215,469,045 social posts were collected for processing, including 8.5 million posts for Mexico and 29 million for the United Kingdom. Table 1 presents the total number of posts collected before classification into specific categories. Twitter data are overrepresented, accounting for 93.31% (188,644,046/202,177,384) of all data. The United States, the United Kingdom, and Brazil accounted for the highest data volumes.

**Table 1:** Total number of data collected per country.

Country	Total posts (N=202,177,384), n (%)	Twitter (n=188,644,046; 93.31%), n (%)	Web content (n=12,208,477; 6.04%), n (%)	Facebook (n=1,324,861; 0.66%), n (%)
Angola	77,666 (100)	39,440 (50.78)	37,349 (48.09)	877 (1.13)
Brazil	21,361,298 (100)	21,120,334 (98.87)	198,439 (0.93)	42,525 (0.2)
Canada	13,521,028 (100)	12,554,897 (92.85)	898,629 (6.65)	67,502 (0.5)
Colombia	3,367,877 (100)	3,239,763 (96.2)	122,244 (3.63)	5870 (0.17)
Democratic Republic of Congo	185,019 (100)	180,488 (97.55)	3731 (2.02)	800 (0.43)
Egypt	745,004 (100)	377,304 (50.64)	339,765 (45.61)	27,935 (3.75)
France	8,754,682 (100)	8,379,214 (95.71)	274,578 (3.14)	100,890 (1.15)
India	10,539,028 (100)	9,876,409 (93.71)	560,071 (5.31)	102,548 (0.97)
Indonesia	4,930,431 (100)	4,681,810 (94.96)	220,653 (4.48)	27,968 (0.57)
Iraq	352,335 (100)	207,738 (58.96)	130,138 (36.94)	14,459 (4.1)
Jordan	301,000 (100)	173,455 (57.63)	93,404 (31.03)	34,141 (11.34)
Kenya	666,320 (100)	587,124 (88.11)	67,273 (10.10)	11,923 (1.79)
Malaysia	2,308,285 (100)	1,607,409 (69.64)	674,606 (29.23)	26,270 (1.14)
Malta	149,348 (100)	74,474 (49.87)	68,065 (45.57)	6809 (4.56)
Mexico	8,542,827 (100)	8,216,404 (96.18)	310,731 (3.64)	15,692 (0.18)
Morocco	293,093 (100)	159,184 (54.31)	127,842 (43.62)	6067 (2.07)
Nicaragua	134,175 (100)	124,389 (92.71)	9062 (6.75)	724 (0.54)
Nigeria	1,203,045 (100)	1,048,254 (87.13)	115,877 (9.63)	38,914 (3.23)
Peru	1,696,642 (100)	1,339,361 (78.94)	345,481 (20.36)	11,800 (0.7)
Philippines	2,116,945 (100)	1,641,901 (77.56)	446,545 (21.09)	28,499 (1.35)
Senegal	142,566 (100)	108,810 (76.32)	24,596 (17.25)	9160 (6.43)
South Africa	3,057,165 (100)	2,697,841 (88.25)	237,888 (7.78)	121,436 (3.97)
Spain	8,904,426 (100)	8,576,075 (96.31)	266,925 (3)	61,426 (0.69)
Switzerland	985,164 (100)	954,406 (96.88)	21,744 (2.21)	9014 (0.91)
Thailand	1,488,541 (100)	1,351,646 (90.80)	95,071 (6.39)	41,824 (2.81)
Trinidad y Tobago	162,215 (100)	140,189 (86.42)	20,652 (12.73)	1374 (0.85)
The United Kingdom	28,955,804 (100)	25,641,890 (88.56)	3,218,910 (11.12)	95,004 (0.33)
The United States	75,975,581 (100)	72,497,816 (95.42)	3,088,538 (4.07)	389,227 (0.51)
Uruguay	1,066,035 (100)	941,735 (88.34)	114,272 (10.72)	10,028 (0.94)
Yemen	193,839 (100)	104,286 (53.8)	75,398 (38.9)	14,155 (7.3)

### Taxonomy Classification

Once the data were collected, 34.44% (74,214,770/215,469,045) were categorized into the taxonomy categories, with the rest dismissed as not carrying relevant information or being considered as noise. To test the ability of the algorithm, 3888 social media posts were sourced from both the Spanish and English data sets. For Spanish language, this was an average of 80.9 (SD 65) texts per category, and for English, 86.8 (SD 44.4) texts per category. Precision, recall, and *F*_1_-score are shown as percentages in Table 2 for all categories. We can see that the machine learning approach outperforms the Boolean query for both languages. The Hotelling *T*^2^ test demonstrated a statistically significant difference between the 2 categorization methods for both languages (*P*≤.001).

The largest difference was observed in precision, with a 16-point difference in English, and a 9.5-point difference for Spanish. The machine learning algorithm disambiguates the meaning of some words that are not possible using Boolean queries. Table 3 shows some examples in which the algorithm correctly categorizes the text.

The same calculations were run for each category, with the full results available in Multimedia Appendix 5. We found that the machine learning algorithm outperformed the Boolean query method for 80% (33/41) of categories for recall, 88% (36/41) for precision, and 93% (38/41) for *F*_1_-scores for the English language. For Spanish, there was a better recall (26/41, 63%), better precision (34/41, 83%), and better *F*_1_-scores (32/41, 78%). Globally, the results were better for the English language when compared with Spanish. The *F*_1_-score was 20 points higher for English than that for Spanish.

**Table 2:** Precision and recall for algorithm versus Boolean methods per language.

Language and model			Precision (%)		Recall (%)		*F*_1_-score (%)		
**English**									
	Algorithm	67.54		81.57		73.90			
	Boolean	51.45		74.75		60.95			
**Spanish**									
	Algorithm	45.28		64.79		53.30			
	Boolean	35.77		62.36		45.46			

**Table 3:** Examples of categorization errors using Boolean query that are well predicted by the semisupervised algorithm.

Input	Algorithm			
	Semisupervised learning		Boolean queries	
	Category predicted	Result	Category predicted	Result
*r aware of 666 is name written in da bible generated frm da word corona now you know antichrist NEED God almighty intervene n manifest Himself only God can save the world from falsehood n demonic china virus*	Faith	True positive	The Cause of the Virus	False positive
*Our Summary Report is your essential guide to update data in the most promising markets, covering:* Global market stats; Market intel on key MENA and SEA countries; Hot topics like online schools & the impact of COVID-19. *Order a copy*	Statistics & Data	True positive	Transmission Settings	False positive
‘*What, i rarely test myself xx like today spoke with a workmate said a had a sore throat, ear ache and he said take a test, i am like No its not covid’*	Other Discussed Symptoms	True positive	Testing	False positive

### Demographics and Intent

The gender split of data gathered for the EARS platform was largely consistent with population-level data on social media use from Hootsuite [37,38]. Table 4 presents a comparison of the gender proportion output from EARS using the demographics segmentation algorithm across 2 countries, the United Kingdom and Mexico, for 2021 compared with Hootsuite data from surveys on social media in the same year.

**Figure 2:**
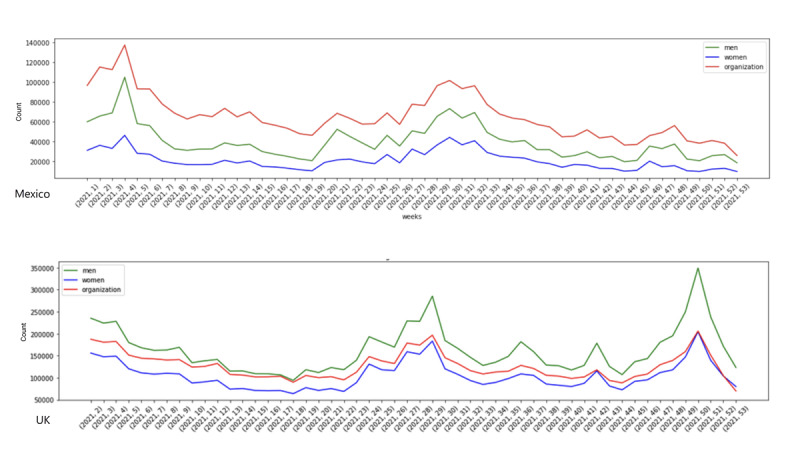
Number of social media posts per week over time in Mexico, (top) and United Kingdom (bottom) shown by those posted by men (green), women (blue) and organizations (red).

For both countries, we mapped the number of social media posts by demographic segment for Mexico and in the United Kingdom (Figure 2). In Mexico, organizations made a higher number of posts than those from either men or women. Data from institutions represented almost half of the discussions in 2021. In the United Kingdom, data from men were more highly represented than those from women by more than 100,000 posts per week. Institutions were also more highly represented than women. We can see from the data that posting patterns are largely similar across demographic types.

**Table 4:** Gender proportion of social media users in Early Artificial Intelligence–Supported Response with Social Listening (EARS) data set, compared with Hootsuite country-level data.

Country and gender		EARS, n (%)	Hootsuite (%)
**Mexico (n=3,961,325 posts)**			
	Men	2,574,861 (65)	61.1
	Women	1,386,463 (35)	38.9
**The United Kingdom (n=17,311,716 posts)**			
	Men	10,508,211 (60.7)	60.1
	Women	6,803,504 (39.3)	39.9

### Category Analysis

Velocity data for Mexico and the United Kingdom were included. Table 5 lists the categories with the highest number of velocity alerts between December 2020 and February 2022. These data were differentiated by gender. Data from all categories over this time are included in Multimedia Appendix 6. The mean number of velocity alerts across all categories in the aggregate is 28.96 for Mexico and 26.67 for the United Kingdom.

The top 3 categories for Mexico were “Youth” (39 weeks with velocity higher than 15%), “General Vaccine Discussion” (38 weeks with velocity higher than 15%), and “Modes of Transmission” (37 weeks with velocity higher than 15%). For the United Kingdom, the categories with the highest velocity alerts were “Immunity” (40 weeks with velocity higher than 15%), “Modes of Transmission” (35 weeks with velocity higher than 15%), and “Civil Unrest” (34 weeks with velocity higher than 15%). In Mexico, there are differences in category velocity between genders. For women, there were high increases in “Modes of Transmission” (21 weeks with velocity higher than 15%), “Youth” (20 weeks with velocity higher than 15%) and “General Vaccine Discussion” (19 weeks with velocity higher than 15%), whereas for men, “Statistics & Data” (19 weeks with velocity higher than 15%), “Youth” (19 weeks with velocity higher than 15%), and “General Vaccine Distinction” (19 weeks with velocity higher than 15%) were the highest. In the United Kingdom, the top velocity categories for men in 2021 were “Immunity” (21 weeks with velocity higher than 15%), “Transmission Settings” (19 weeks with velocity higher than 15%), and “Health Care Workers & Vaccine” (19 weeks with velocity higher than 15%), whereas for women, it was “Immunity” (19 weeks with velocity higher than 15%), “Modes of Transmission” (18 weeks with velocity higher than 15%), and “Civil Unrest” (18 weeks with velocity higher than 15%).

Data can also be filtered by post intent, that is, whether it is a questioning post. Combining these filters—the taxonomy categories, the demographics segments, and the query detector—helps in the quantitative analysis of narrative change. Table 6 presents the number of weeks with a velocity alert for questioning posts for men and women in the United Kingdom and Mexico. These data were restricted to individuals (not institutions). The list of all category velocity changes by question for this time is included in Multimedia Appendix 7. The mean number of velocity alerts across all categories for social media posts carrying questions, for both genders, is 31.83 Mexico and 23.2, the United Kingdom.

Table 6 provides valuable insights into which topics have suddenly raised more questions between December 2020 and February 2022. In Mexico, “Other Discussed Symptoms” (45 weeks with velocity higher than 15% for both genders combined), “Measures in Public Settings” (42 weeks with velocity higher than 15% for both genders combined), and “Vaccine Distribution and Policies on Access” (42 weeks with velocity higher than 15% for both genders combined) generated the most velocity alerts. In the United Kingdom, velocity alerts were oriented toward “Health Technology” (40 weeks with velocity >15% for both genders combined), “Digital Health Technology” (39 weeks with velocity higher than 15% for both genders combined), and “Other Discussed Symptoms” (39 weeks with velocity higher than 15% for both genders combined).

This level of data filtering also provides more visibility by gender in specific areas of concern. In Mexico, the highest velocity question change for men was “General Vaccine Discussion” (23 weeks with velocity higher than 15% for both genders combined) and “Immunity” (22 weeks with velocity higher than 15% for both genders combined), whereas for women, it was “Myths” (22 weeks with velocity higher than 15% for both genders combined) and “Supportive Care” (21 weeks with velocity higher than 15% for both genders combined). In the United Kingdom, the highest number of changes for men was “Pandemic Fatigue” (18 weeks with velocity higher than 15% for both genders combined), whereas for women, it was “Stigma about the Spread” (18 weeks with velocity higher than 15% for both genders combined).

The table also highlights that some topics raised many more questions at certain points for men than for women and vice versa. For instance, in Mexico, “Pandemic Fatigue” raised twice the number of velocity alerts for men than for women, whereas the “Current Treatments” topic raised 6 more weekly velocity alerts for women. In the United Kingdom, “Reduction of Domestic Movement” was of higher concern for men, whereas “Faith” and “Civic Unrest” were higher among women.

The monitoring system provided valuable insights throughout the pandemic by providing analysts with information to inform further investigations and focus on attention. This included narrative identification at the country level regarding concerns about vaccine side effects; questions about the cause of the virus at different time points, and particularly early on in the pandemic; and concerns and questions about specific measures introduced to reduce movement.

**Table 5:** Number of velocities alerts by category and gender from December 2020 to February 2022.

Categories	Mexico, n (%)				The United Kingdom, n (%)		
	Total number of velocity alerts	Velocity alerts by gender		Total number of velocity alerts		Velocity alerts by gender	
		Men	Women			Men	Women
Youth	39 (100)	19 (49)	20 (51)	30 (100)		16 (53)	14 (47)
General vaccine discussion	38 (100)	19 (50)	19 (50)	30 (100)		15 (50)	15 (50)
Modes of transmission	37 (100)	16 (43)	21 (57)	35 (100)		17 (49)	18 (51)
Statistics and data	33 (100)	19 (58)	14 (42)	30 (100)		15 (50)	15 (50)
Transmission settings	31 (100)	17 (55)	14 (45)	33 (100)		18 (55)	15 (45)
Pandemic fatigue	31 (100)	18 (58)	13 (42)	24 (100)		13 (54)	11 (46)
Health care workers and vaccine	31 (100)	16 (52)	15 (48)	32 (100)		18 (56)	14 (44)
Digital health technology	30 (100)	13 (43)	17 (57)	20 (100)		11 (55)	9 (45)
Civil unrest	28 (100)	14 (50)	14 (50)	34 (100)		16 (47)	18 (53)
Immunity	36 (100)	18 (50)	18 (50)	40 (100)		21 (52)	19 (48)

**Table 6:** Number of velocities alerts by category question rate and gender from December 2020 to February 2022.

Categories	Mexico, n (%)				The United Kingdom, n (%)		
	Total number of velocity alerts in questions	Velocity alerts by gender—questions		Total number of velocity alerts in questions		Velocity alerts by gender—questions	
		Men	Women			Men	Women
Other discussed symptoms	45 (100)	25 (56)	20 (44)	35 (100)		18 (51)	17 (49)
Measures in public settings	42 (100)	21 (50)	21 (50)	22 (100)		10 (45)	12 (55)
Vaccine distribution and policies on access	42 (100)	21 (50)	21 (50)	21 (100)		8 (38)	13 (62)
Myths	42 (100)	20 (48)	22 (52)	25 (100)		10 (40)	15 (60)
Digital health technology	40 (100)	19 (48)	21 (52)	39 (100)		21 (54)	18 (46)
Faith	40 (100)	20 (50)	20 (50)	25 (100)		8 (32)	17 (68)
General vaccine discussion	39 (100)	23 (59)	16 (41)	23 (100)		10 (43)	13 (57)
Health technology	39 (100)	19 (49)	20 (51)	40 (100)		19 (48)	21 (52)
Supportive care	38 (100)	17 (45)	21 (55)	18 (100)		9 (50)	9 (50)
Immunity	39 (100)	22 (56)	17 (44)	20 (100)		9 (45)	11 (55)
Stigma around the spread	32 (100)	16 (50)	16 (50)	30 (100)		12 (40)	18 (60)
Civil unrest	32 (100)	16 (50)	16 (50)	30 (100)		12 (40)	18 (60)
Pandemic fatigue	30 (100)	20 (67)	10 (33)	34 (100)		18 (53)	16 (47)
Reduction of domestic movement	29 (100)	13 (45)	16 (55)	18 (100)		13 (72)	5 (28)
Current treatments	26 (100)	10 (38)	16 (62)	24 (100)		10 (42)	14 (58)

## Discussion

### Principal Findings

The configuration and application of the EARS platform have enabled WHO infodemic managers to access categorized data throughout the pandemic to inform responses.

Compared with other analytical methods that are more manual, required data scientists in the team, or had fewer analytics capabilities, the EARS platform has enabled progress toward more scalable and sustainable analysis of social media for public health action. Real-time data collection and categorization were fully automated, enabling a self-serving model of use. This study has demonstrated the strength of a machine learning algorithm for categorizing COVID-19 narratives into a public health taxonomy. The proposed approach outperformed the Boolean query strategy across all metrics. This approach has not only allowed data to be quickly categorized and useful for infodemic managers but also to change and grow as conversations shift. The algorithm disambiguates the meaning of some words considering co-occurrences and other words in close proximity, considering the context around the keywords rather than just the word. This has enabled the algorithm to include new and emerging words and phrases, which is essential as the infodemic around COVID-19 has moved at such a pace. However, human inputs are still needed for contextualization and translation into actionable insights. The separation of the overall global conversation into country-level, category-specific, intent-specific, and demographically segmented analytics is a significant step toward obtaining more usable and useful data for decision-making.

Other studies have combined machine learning and COVID-19 pandemic data, mainly focusing on 1 component of the information analysis. In 1 study, NLP bots were trained to detect misinformation on the Reddit platform by fine-tuning the BERT model [40]. Another United Kingdom–based study applied sentiment analysis on COVID-19 mental health–related tweets [41]. An NLP study developed to recognize COVID-19 symptoms described in social media posts and used them for disease surveillance and detection reported useful data to identify the prevalence and severity of the symptoms [42]. Other studies explored vaccine-related tweets to identify vaccination-related topics [43,44]. This paper has reported on the categorization of all COVID-19–related narratives, across multiple languages and countries, and adds to the evidence on how infodemic insights can be identified during a health emergency.

The methodology described herein has several strengths. Most social media analysis research is conducted in high-income countries [2], and research and tools that focus on low- and middle-income countries are required. Table 2 presents how overrepresented the American and European regions are in this data set, accounting for 85% of all data collected. By allowing for filtering at the regional and country levels, we can prioritize narratives from other regions to better understand global trends. Although the limitation—that smaller topics of conversation that are gathering velocity and volume are “drowned out” by the major narratives—has been partially addressed by showing relative importance across countries on smaller narratives, more needs to be done on emerging topic detection of topics and increasing diversity of data sources. The language-agnostic approach has enabled scaling and addition of more languages. Filtering by questions and gender allows analysts to quickly find meaning in the data.

Moreover, recent proposals for amendments to International Health Regulations emphasize the need to expand the use of digital applications for public health and call for increasing country infrastructure and human capacities to use technology to support public health [45,46]. This highlights the increased burden that individual infodemics have placed on emergency response structures and health systems and requires intensified investment in systematic strategies for infodemic management.

There are clear areas for future research and work. The area of network analysis to identify narrative amplifiers (eg, users or communities sharing misinformation narratives or sharing information across thematic and interest communities) or social structures about how information flows in the network is a promising tool for infodemic managers. However, this approach would need to carefully balance the potential trade-off between valuable actionable information and user privacy. At an aggregated level, further segmentation of groups can be accomplished (such as studying conversations by health care professionals or disaggregating organizational content). The machine learning tool, along with demographic segmentation, helps gain insights from data trends. Knowing where questions are rising, for example, can help to identify information voids and opportunities to target public health advice and information materials.

Although digital social media analysis platforms are an important part of understanding community perceptions and concerns, an integrated analysis of infodemic insights, including the combination of offline and web-based sources to triangulate data, is needed [12]. In addition to expanding the data sets in EARS to include more diverse data, there will always be limitations in infodemic insights data sources from digital platforms and web-based public conversations, making integrated analysis a vital and necessary step in the generation of infodemic insights and recommendations for action. Although the EARS dashboards can provide rich data and “signal” to analysts, human interpretation is needed to contextualize and translate this into actionable insights.

### Limitations

This study has some limitations. The machine learning model assumes mutual exclusion for categories and only enables mono-label predictions. This means that we predicted only one category for each text. Conversations about COVID-19 may contain threads that cross several categories, and the inability to multitag data across categories may result in data meaning being lost from analysis within categories that would be relevant. A principal path of improvement would be to extend the model to multilabel classification.

As noted in previous research, the analysis of web-based conversations in academic and marketing sector analytics continues to have overreliance on Twitter data, which has limited representativity even if we can make demographics identification to limit the biases. This is also the case for the EARS platform. The platform will continue to gradually add more data sources, which can expand its coverage and representativity. In addition, comparing with non–text-based sources can help increase representativity, as well as provide a more rounded view of the data. Integrating fact-checker and misinformation data sets could yield more insight into the drivers and effects of the infodemic in the digital data sources. EARS provides a public health–relevant tool for the analysis of digital and web-based data sources in several languages and therefore improves the analysis of data from internet platforms. However, insights that are gleaned from EARS must still be integrated with intelligence from other data sources that cover communities, behaviors, users, and information seeking to inform infodemic management strategies.

The categorization rate of 37% means that most data identified as COVID-19 related is uncategorized. This limitation is addressed by a regular review of uncategorized data and updating of seed words. Although geo-located data are required for country-level analysis, this limits the amount of data that can be included. Gender data were currently disaggregated by men and women only. This is a limitation in interpreting the views of those who were not identified as men or women. The platform currently does not segment by age, which is another planned addition.

### Conclusions

The WHO EARS platform described here has been specifically developed to address the changing needs of public health analysts during the COVID-19 pandemic. The platform was designed to allow scalability and iterations, and new countries and languages have been added. The platform’s digital architecture has overcome many of the challenges inherent in social media analysis; however, more work is still required. An integrated analysis of other data sources, including offline sources, is still needed, and the implementation of multilabel classification will further aid analysts. Although much of the categorization is automated, human analysts are still needed to contextualize and triangulate the data to create actionable insights.

The application of a public health taxonomy and AI technology to a user-friendly social listening platform, accessible directly by analysts, is a significant step toward a better understanding of global narratives. The scalability and rounds of review and iteration mean that the platform will continue to evolve throughout the pandemic and respond to user needed. As new global emergencies emerge, a key challenge will be remaining agile enough to pivot as needed and incorporate emerging trends.

The WHO EARS platform has applied novel analytic approaches to improve the generation of infodemic intelligence and, therefore, strengthen the evidence base for infodemic management. As the platform matures, there is an increased opportunity for countries to adopt it, or similar tools, in infodemic insight analysis.

## Appendix

Multimedia Appendix 1 Early Artificial Intelligence–Supported Response With Social Listening (EARS) COVID-19 taxonomy categories and definition.

Multimedia Appendix 2 Pilot countries.

Multimedia Appendix 3 COVID-19 keywords and queries to collect social media posts for each country.

Multimedia Appendix 4 English and Spanish Keywords for each individual category.

Multimedia Appendix 5 Precision and recall metrics for algorithm versus Boolean methods for all categories.

Multimedia Appendix 6 Number of velocities alerts by category from December 2020 to February 2022.

Multimedia Appendix 7 Number of velocities alerts by category and question filter from December 2020 to February 2022.

## Abbreviations

AI: artificial intelligence
BERT: bidirectional encoder representations from transformers
EARS: Early Artificial Intelligence–Supported Response With Social Listening
FP: false positive
NLP: natural language processing
TP: true positive
WHO: World Health Organization
