# Supplementary material for: Using Machine Learning Technology (Early Artificial Intelligence–Supported Response With Social Listening Platform) to Enhance Digital Social Understanding for the COVID-19 Infodemic: Development and Implementation Study
Source: JMIR Infodemiology. 2023 Aug 21;3:e47317. doi: 10.2196/47317 (PMC10477919; doi:10.2196/47317)
Supplement: Multimedia Appendix 1 [file infodemiology_v3i1e47317_app1.docx]

**Multimedia Appendix 1. EARS COVID-19 taxonomy categories and definition**

**The cause: How did the virus emerge and how is it spreading?**

1. The cause of the virus: Narratives about the origin of SARS-CoV-2.
2. Stigma about the spread: Stigma on people who are thought of spreading the virus: racist expressions, attribution to poor people or immigrants.
3. Stigma about or by infected people: Stigma expressed about or by infected people or have been infected.

**The illness: What are the symptoms and how is it transmitted?**

1. Confirmed symptoms: Confirmed symptoms as defined by WHO, excluding longer-term symptoms.
2. Other discussed symptoms: Other discussed symptoms that have not yet been confirmed by WHO.
3. Prolonged symptoms: Reports on long covid that may or may not be confirmed by WHO.
4. Modes of transmission: Modes of transmission confirmed and unconfirmed by WHO. This includes discussion of asymptomatic and pre-symptomatic transmission as well as possible ways the virus can be transmitted (for example, aerosols and fomites).
5. Transmission settings: Narratives about settings where transmission can be amplified: closed and semi-closed settings.
6. Immunity: General conversations on re-infection, confusion over immunity after infection or the possibility of being infected more than once.
7. COVID 19 Variants: Narratives and concerns about the development, spread and impact of new COVID 19 Variants.
8. Demographic vulnerability & risks: Vulnerable and risk groups are the elderly, individuals with individuals with health conditions like lung or heart disease, diabetes or conditions that affect their immune system, pregnant women
9. Impact on mental health: Anxiety, depression and other affections derived from the pandemic situation

**The treatment: How can it be treated or cured?**

1. Current treatment*: Medical treatment as per WHO treatment recommendations
2. COVID-19 vaccine: Narratives about the vaccine itself: efficacy, side effects, safety, etc.
3. Health care workers (HCW) and vaccine: Narratives by and about health care workers and vaccine
4. General vaccine discussion. Narratives about vaccines in general, including discussion about others or communities that have different opinions about vaccines; can include any vaccine concerns, not just COVID-19
5. Science and R&D: Comments on new treatment and vaccines from research and development and evidence and scientific processes
6. Non proven treatments: Discussion about treatments that are not proven to be effective (examples: sunlight, nutrition, herbal remedies, etc)
7. Myths: Specific myths that WHO and partners have reacted to taken steps to debunk reference

**The interventions: What is being done by government and health authorities and societal institutions?**

1. Testing: Any discussion about tests – everything from reliability, to access to tests, types of tests, requirement to have tests, etc.
2. Contact tracing: Any discussion about the process, requirements and steps involved in contact tracing, use of technology
3. Supportive care: Care given to patients in hospitals by medical personnel
4. Vaccine distribution and policies on access: Narratives about distribution, equity, access to COVID-19 vaccine
5. Personal measures: Individual protection measures recommended by governments/WHO such as wearing masks, handwashing, social distance, isolation when ill…
6. Measures in public settings: Measures implemented by governments in public settings: schools, workplaces, public transport…
7. Travel measures: Measures implemented or suggested by governments/WHO/population/private companies on travel: negative PCR or negative rapid test to enter a country, mandatory quarantine
8. Immunity pass: Vaccine certificates, immunity / health passports, digital and hard copy, including implications for access to businesses, schools, and other services.
9. Reduction of movement: Measures implemented by governments related to movement reduction: lock-down at home, territory lock-down, etc.
10. Protection: medical equipment: Equipment for health workers: PPE advances and accessibility for the public.
11. Health Technology: Health technology used to treat patients: medicines, medical devices, vaccines, procedures and systems
12. Digital health technology: Discussions about digital technology used to respond to pandemic: electronic data exchange, electronic notices of passenger lists to health authorities, biometric data coming from wearables, proximity apps (App Covid). Includes people’s attitudes to data privacy, or for modelling and predictive analytics.
13. Pandemic Fatigue: Fatigue from interventions (lock-down, movement restrictions, masks...)
14. Faith: Narratives about faith and religion and COVID-19 (these narratives are recurring, usually around the time of religious holidays and outbreaks in faith based settings)
15. Industry & economy Narratives about industry, unions and COVID-19 and how the pandemic has affected the economy.
16. Environment: Narratives about the environment and COVID-19 – some examples: shading in environment, waste water, air pollution as a secondary byproduct of lockdowns
17. Inequalities & Human Rights: Narratives about social inequalities and relation to COVID-19
18. Civil Unrest: Narratives about civil unrest provoked or associated with the pandemic and its restrictions.
19. Youth: Narratives about youth, effects of pandemic on them, or actions youth is taking

**Type of information: What types of information are most engaging?**

1. Statistics & data Conversations about facts, official statistics and data
2. Mis- and Disinformation: Conversations, attitudes and perceptions about mis- and disinformation
3. Sources & influencers: Conversations about where people look for information
