# Supplementary material for: Using Machine Learning Technology (Early Artificial Intelligence–Supported Response With Social Listening Platform) to Enhance Digital Social Understanding for the COVID-19 Infodemic: Development and Implementation Study
Source: JMIR Infodemiology. 2023 Aug 21;3:e47317. doi: 10.2196/47317 (PMC10477919; doi:10.2196/47317)
Supplement: Multimedia Appendix 2 [file infodemiology_v3i1e47317_app2.docx]

**Multimedia Appendix 2. Pilot countries**

| 1. Angola 2. Brazil 3. Canada 4. Colombia 5. Democratic Republic of the Congo 6. Egypt 7. France 8. India 9. Indonesia 10. Iraq 11. Jordan 12. Kenya 13. Malaysia 14. Malta 15. Mexico | 1. Morocco 2. Nicaragua 3. Nigeria 4. Peru 5. Philippines 6. Senegal 7. South Africa 8. Spain 9. Switzerland 10. Thailand 11. The United Kingdom 12. Trinidad and Tobago 13. United States of America 14. Uruguay 15. Yemen |
| --- | --- |
