# Supplementary material for: Using Machine Learning Technology (Early Artificial Intelligence–Supported Response With Social Listening Platform) to Enhance Digital Social Understanding for the COVID-19 Infodemic: Development and Implementation Study
Source: JMIR Infodemiology. 2023 Aug 21;3:e47317. doi: 10.2196/47317 (PMC10477919; doi:10.2196/47317)
Supplement: Multimedia Appendix 3 [file infodemiology_v3i1e47317_app3.docx]

**Multimedia Appendix 3. COVID-19 keywords and queries to collect social media posts for each country**

**List of queries for each country**

| **Country** | **Language** | **Query** |
| --- | --- | --- |
| Angola | Portuguese,  English, Spanish | (profile_country:AO (covid OR covid19 OR covid-19 OR covid_19 OR corona OR coronavirus OR virus OR nCoV OR SARSCoV2 OR infecção OR infectado OR infectada OR infecções OR contágio OR contagioso OR contagiosa OR pandemia OR "terceira onda" OR "quarta onda" OR variante OR vacina OR vacinas OR vacinação OR vacinada OR vacinada OR infección OR infectado OR infectada OR infecciones OR contagio OR contagioso OR contagiado OR contagiada OR contagios OR pandemia OR "tercera ola" OR "cuarta ola" OR variante OR vacuna OR vacunas OR vacunación OR vacunado OR vacunada OR MINSA OR OMS OR @WHO OR @AngolaOms) OR (Angola)(covid OR corona OR vacina OR vacinação OR OMS) - (is:verified OR RT OR cerveja OR ações OR futebol OR partido OR eleições OR votos OR voto OR eleitores OR "novos casos" OR "casos confirmados" OR "novos casos" OR "total de casos" OR "total de mortes") |
| Mexico | English, Spanish | profile_country:MX (covid OR covid19 OR covid-19 OR covid_19 OR corona OR coronavirus OR virus OR nCoV OR SARSCoV2 OR infección OR infectado OR infectada OR infecciones OR contagio OR contagioso OR contagiado OR contagiada OR contagios OR pandemia OR "tercera ola" OR "cuarta ola" OR variante OR vacuna OR vacunas OR vacunación OR vacunado OR vacunada OR Pfizer OR Moderna OR AstraZeneca OR AZ OR J&J OR Janssen OR "Sputnik V" OR Sinovac OR Novavax OR Curevax OR Covax OR @SSalud_mx OR OMS OR @WHO OR @OPSOMSMexico) - (is:verified OR RT OR cerveza OR acciones OR fútbol OR partido OR elecciones OR votos OR voto OR votantes OR "nuevos casos" OR "casos confirmados" OR "casos nuevos" OR "casos totales" OR "muertes totales") |
| Brazil | English, Portuguese | profile_country:BR (covid OR covid19 OR covid-19 OR covid_19 OR corona OR coronavirus OR virus OR nCoV OR SARSCoV2 OR infecção OR infectado OR infectada OR infecções OR contágio OR contagioso OR contagiosa OR pandemia OR "terceira onda" OR "quarta onda" OR variante OR vacina OR vacinas OR vacinação OR vacinada OR vacinada OR Pfizer OR Moderna OR AstraZeneca OR AZ OR J&J OR Janssen OR "Sputnik V" OR Sinovac OR Novavax OR Curevax OR Covax OR @minsaude OR OMS OR @WHO OR @OPASOMSBrasil) - (is:verified OR RT OR cerveja OR ações OR futebol OR partido OR eleições OR votos OR voto OR eleitores OR "novos casos" OR "casos confirmados" OR "novos casos" OR "total de casos" OR "total de mortes") |
| Canada (EN) | English | profile_country:CA (lang:en) ((covid OR covid19 OR covid-19 OR covid_19 OR corona OR coronavirus OR virus OR nCoV OR SARSCoV2 OR infection OR infected OR infections OR contagion OR contagious OR contagions OR pandemic OR "3rd wave" OR "4th wave" OR "infection curve" OR variant OR vaccine OR jab OR vaccines OR vaccinated OR vaccination OR Pfizer OR Moderna OR AstraZeneca OR AZ OR J&J OR Janssen OR "Sputnik V" OR Sinovac OR Novavax OR Curevax OR Covax OR @GovCanHealth OR @WHO) - (is:verified OR RT OR beer OR stocks OR football OR game OR cricket OR elections OR votes OR voters OR vote OR "new cases" OR "total cases" OR "total deaths") |
| Colombia | English, Spanish | profile_country:CO (covid OR covid19 OR covid-19 OR covid_19 OR corona OR coronavirus OR virus OR nCoV OR SARSCoV2 OR infección OR infectado OR infectada OR infecciones OR contagio OR contagioso OR contagiado OR contagiada OR contagios OR pandemia OR "tercera ola" OR "cuarta ola" OR variante OR vacuna OR vacunas OR vacunación OR vacunado OR vacunada OR Pfizer OR Moderna OR AstraZeneca OR AZ OR J&J OR Janssen OR "Sputnik V" OR Sinovac OR Novavax OR Curevax OR Covax OR @MinSaludCol OR OMS OR @WHO OR @OPSOMS_Col) - (is:verified OR RT OR cerveza OR acciones OR fútbol OR partido OR elecciones OR votos OR voto OR votantes OR "nuevos casos" OR "casos confirmados" OR "casos nuevos" OR "casos totales" OR "muertes totales") |
| Congo DRC | English, French | (lang:fr OR lang:en)(((profile_country:CD) (covid OR covid19 OR covid-19 OR covid_19 OR corona OR coronavirus OR virus OR nCoV OR SARSCoV2 OR infecté OR infectée OR infectées OR contagiuex OR contagion OR pandemie OR "troisieme vague" OR "quatrieme vague" OR variant OR vaccin OR vaccins OR vaccination OR vacciné OR vaccinée OR Pfizer OR Moderna OR AstraZeneca OR AZ OR J&J OR Janssen OR "Sputnik V" OR Sinovac OR Novavax OR Curevax OR Covax OR @MinSanteRDC OR @OMSRDCONGO OR OMS OR @WHO)) OR (@MinSanteRDC OR @OMSRDCONGO) OR (Congo OR DRC)(Covid OR coronavirus OR corona OR pandemie OR vaccin OR vacciné OR OMS OR @WHO OR Covax)) - (is:verified OR RT OR beer OR stocks OR football OR game OR cricket OR elections OR votes OR voters OR vote OR "new cases" OR "total cases" OR "total deaths" OR bière OR bouse OR match OR elections OR votes OR votants OR electeurs OR "nouveaux cas" OR "nombre de décès" OR "nombre de cas") |
| France | English, French | profile_country:FR (covid OR covid19 OR covid-19 OR covid_19 OR corona OR coronavirus OR virus OR nCoV OR SARSCoV2 OR infecté OR infectée OR infectées OR contagiuex OR contagion OR pandemie OR "troisieme vague" OR "quatrieme vague" OR variant OR vaccin OR vaccins OR vaccination OR vacciné OR vaccinée OR Pfizer OR Moderna OR AstraZeneca OR AZ OR J&J OR Janssen OR "Sputnik V" OR Sinovac OR Novavax OR Curevax OR Covax OR @SantePubliqueFr OR OMS OR @WHO OR @WHO_Europe) - (is:verified OR RT OR beer OR stocks OR football OR game OR cricket OR elections OR votes OR voters OR vote OR "new cases" OR "total cases" OR "total deaths" OR bière OR bouse OR match OR elections OR votes OR votants OR electeurs OR "nouveaux cas" OR "nombre de décès" OR "nombre de cas") |
| India | English | profile_country:IN (lang:en) (covid OR covid19 OR covid-19 OR covid_19 OR corona OR coronavirus OR virus OR nCoV OR SARSCoV2 OR infection OR infected OR infections OR contagion OR contagious OR contagions OR pandemic OR "3rd wave" OR "4th wave" OR "infection curve" OR variant OR vaccine OR jab OR vaccines OR vaccinated OR vaccination OR Pfizer OR Moderna OR AstraZeneca OR AZ OR J&J OR Janssen OR "Sputnik V" OR Sinovac OR Novavax OR Curevax OR Covax OR @MoHFW_INDIA OR @WHO OR @WHOSEARO) - (is:verified OR RT OR beer OR stocks OR football OR game OR cricket OR elections OR votes OR voters OR vote OR "new cases" OR "total cases" OR "total deaths") |
| Indonesia | English | profile_country:ID (covid OR covid19 OR covid-19 OR covid_19 OR corona OR coronavirus OR virus OR nCoV OR SARSCoV2 OR infection OR infected OR infections OR contagion OR contagious OR contagions OR pandemic OR "3rd wave" OR "4th wave" OR "infection curve" OR variant OR vaccine OR jab OR vaccines OR vaccinated OR vaccination OR infeksi OR terinfeksi OR infeksi OR penularan OR menular OR penularan OR pandemi OR "gelombang ke-3" OR "gelombang ke-4" OR "kurva infeksi" OR varian OR vaksin OR suntikan OR vaksin OR divaksinasi OR vaksinasi OR Pfizer OR Moderna OR AstraZeneca OR AZ OR J&J OR Janssen OR "Sputnik V" OR Sinovac OR Novavax OR Curevax OR Covax OR @WHO OR @WHOIndonesia) - (is:verified OR RT OR beer OR stocks OR football OR game OR cricket OR elections OR votes OR voters OR vote OR "new cases" OR "total cases" OR "total deaths" OR "kasus baru" OR "jumlah kasus" OR "jumlah kematian") |
| Kenya | English | profile_country:KE ((covid OR covid19 OR covid-19 OR covid_19 OR corona OR coronavirus OR virus OR nCoV OR SARSCoV2 OR infection OR infected OR infections OR contagion OR contagious OR contagions OR pandemic OR "3rd wave" OR "4th wave" OR "infection curve" OR variant OR vaccine OR jab OR vaccines OR vaccinated OR vaccination OR Pfizer OR Moderna OR AstraZeneca OR AZ OR J&J OR Janssen OR "Sputnik V" OR Sinovac OR Novavax OR Curevax OR Covax OR @MOH_Kenya OR @WHO OR @WHOKenya) - (is:verified OR RT OR beer OR stocks OR football OR game OR cricket OR elections OR votes OR voters OR vote OR "new cases" OR "total cases" OR "total deaths") |
| Malta | English | ((profile_country:MT(covid OR covid19 OR covid-19 OR covid_19 OR corona OR coronavirus OR virus OR nCoV OR SARSCoV2 OR infection OR infected OR infections OR contagion OR contagious OR contagions OR pandemic OR "3rd wave" OR "4th wave" OR "infection curve" OR variant OR vaccine OR jab OR vaccines OR vaccinated OR vaccination OR Pfizer OR Moderna OR AstraZeneca OR AZ OR J&J OR Janssen OR "Sputnik V" OR Sinovac OR Novavax OR Curevax OR Covax OR @HPDPmalta OR @WHO OR @WHO_Europe)) OR (@HPDPmalta) OR (Malta)(covid OR corona OR vaccine OR vaccinated OR contagion OR infected OR @WHO OR @HPDPmalta)) - (is:verified OR RT OR beer OR stocks OR football OR game OR cricket OR elections OR votes OR voters OR vote OR "new cases" OR "total cases" OR "total deaths") |
| Nigeria | English | profile_country:NG (covid OR covid19 OR covid-19 OR covid_19 OR corona OR coronavirus OR virus OR nCoV OR SARSCoV2 OR infection OR infected OR infections OR contagion OR contagious OR contagions OR pandemic OR "3rd wave" OR "4th wave" OR "infection curve" OR variant OR vaccine OR jab OR vaccines OR vaccinated OR vaccination OR Pfizer OR Moderna OR AstraZeneca OR AZ OR J&J OR Janssen OR "Sputnik V" OR Sinovac OR Novavax OR Curevax OR Covax OR @Fmohnigeria OR @WHO OR @WHONigeria) - (is:verified OR RT OR beer OR stocks OR football OR game OR cricket OR elections OR votes OR voters OR vote OR "new cases" OR "total cases" OR "total deaths") |
| Phillipines | English | profile_country:PH ((covid OR covid19 OR covid-19 OR covid_19 OR corona OR coronavirus OR virus OR nCoV OR SARSCoV2 OR infection OR infected OR infections OR contagion OR contagious OR contagions OR pandemic OR "3rd wave" OR "4th wave" OR "infection curve" OR variant OR vaccine OR jab OR vaccines OR vaccinated OR vaccination OR Pfizer OR Moderna OR AstraZeneca OR AZ OR J&J OR Janssen OR "Sputnik V" OR Sinovac OR Novavax OR Curevax OR Covax OR @DOHgovph OR @WHO OR @WHOPhilippines) - (is:verified OR RT OR beer OR stocks OR football OR game OR cricket OR elections OR votes OR voters OR vote OR "new cases" OR "total cases" OR "total deaths") |
| Senegal | English, France | profile_country:SN (covid OR covid19 OR covid-19 OR covid_19 OR corona OR coronavirus OR virus OR nCoV OR SARSCoV2 OR infecté OR infectée OR infectées OR contagiuex OR contagion OR pandemie OR "troisieme vague" OR "quatrieme vague" OR variant OR vaccin OR vaccins OR vaccination OR vacciné OR vaccinée OR Pfizer OR Moderna OR AstraZeneca OR AZ OR J&J OR Janssen OR "Sputnik V" OR Sinovac OR Novavax OR Curevax OR Covax OR @sante_gouv_sn OR OMS OR @WHO OR @OMS_SENEGAL) - (is:verified OR RT OR beer OR stocks OR football OR game OR cricket OR elections OR votes OR voters OR vote OR "new cases" OR "total cases" OR "total deaths" OR bière OR bouse OR match OR elections OR votes OR votants OR electeurs OR "nouveaux cas" OR "nombre de décès" OR "nombre de cas") |
| South Africa | English | profile_country:ZA ((covid OR covid19 OR covid-19 OR covid_19 OR corona OR coronavirus OR virus OR nCoV OR SARSCoV2 OR infection OR infected OR infections OR contagion OR contagious OR contagions OR pandemic OR "3rd wave" OR "4th wave" OR "infection curve" OR variant OR vaccine OR jab OR vaccines OR vaccinated OR vaccination OR Pfizer OR Moderna OR AstraZeneca OR AZ OR J&J OR Janssen OR "Sputnik V" OR Sinovac OR Novavax OR Curevax OR Covax OR @HealthZA OR @WHO OR @WHOSouthAfrica) - (is:verified OR RT OR beer OR stocks OR football OR game OR cricket OR elections OR votes OR voters OR vote OR "new cases" OR "total cases" OR "total deaths") |
| Spain | English, Spanish | profile_country:ES (covid OR covid19 OR covid-19 OR covid_19 OR corona OR coronavirus OR virus OR nCoV OR SARSCoV2 OR infección OR infectado OR infectada OR infecciones OR contagio OR contagioso OR contagiado OR contagiada OR contagios OR pandemia OR "tercera ola" OR "cuarta ola" OR variante OR vacuna OR vacunas OR vacunación OR vacunado OR vacunada OR Pfizer OR Moderna OR AstraZeneca OR AZ OR J&J OR Janssen OR "Sputnik V" OR Sinovac OR Novavax OR Curevax OR Covax OR "[Name Minister of Health"] OR @sanidadgob OR OMS OR @WHO OR @WHO_Europe) - (is:verified OR RT OR cerveza OR acciones OR fútbol OR partido OR elecciones OR votos OR voto OR votantes OR "nuevos casos" OR "casos confirmados" OR "casos nuevos" OR "casos totales" OR "muertes totales") |
| Malaysia | English | profile_country:MY ((covid OR covid19 OR covid-19 OR covid_19 OR corona OR coronavirus OR virus OR nCoV OR SARSCoV2 OR infection OR infected OR infections OR contagion OR contagious OR contagions OR pandemic OR "3rd wave" OR "4th wave" OR "infection curve" OR variant OR vaccine OR jab OR vaccines OR vaccinated OR vaccination OR Pfizer OR Moderna OR AstraZeneca OR AZ OR J&J OR Janssen OR "Sputnik V" OR Sinovac OR Novavax OR Curevax OR Covax OR @KKMPutrajaya OR @WHO OR @WHOMalaysia) - (is:verified OR RT OR beer OR stocks OR football OR game OR cricket OR elections OR votes OR voters OR vote OR "new cases" OR "total cases" OR "total deaths") |
| United Kingdom | English | profile_country:GB ((covid OR covid19 OR covid-19 OR covid_19 OR corona OR coronavirus OR virus OR nCoV OR SARSCoV2 OR infection OR infected OR infections OR contagion OR contagious OR contagions OR pandemic OR "3rd wave" OR "4th wave" OR "infection curve" OR variant OR vaccine OR jab OR vaccines OR vaccinated OR vaccination OR Pfizer OR Moderna OR AstraZeneca OR AZ OR J&J OR Janssen OR "Sputnik V" OR Sinovac OR Novavax OR Curevax OR Covax OR NHS OR @NHSuk OR @WHO) - (is:verified OR RT OR beer OR stocks OR football OR game OR cricket OR elections OR votes OR voters OR vote OR "new cases" OR "total cases" OR "total deaths") |
| United States | English | profile_country:US ((covid OR covid19 OR covid-19 OR covid_19 OR corona OR coronavirus OR virus OR nCoV OR SARSCoV2 OR infection OR infected OR infections OR contagion OR contagious OR contagions OR pandemic OR "3rd wave" OR "4th wave" OR "infection curve" OR variant OR vaccine OR jab OR vaccines OR vaccinated OR vaccination OR Pfizer OR Moderna OR AstraZeneca OR AZ OR J&J OR Janssen OR "Sputnik V" OR Sinovac OR Novavax OR Curevax OR Covax OR @CDCgov OR @WHO) - (is:verified OR RT OR beer OR stocks OR football OR game OR cricket OR elections OR votes OR voters OR vote OR "new cases" OR "total cases" OR "total deaths") |
| Uruguay | English, Spanish | profile_country:UY (covid OR covid19 OR covid-19 OR covid_19 OR corona OR coronavirus OR virus OR nCoV OR SARSCoV2 OR infección OR infectado OR infectada OR infecciones OR contagio OR contagioso OR contagiado OR contagiada OR contagios OR pandemia OR "tercera ola" OR "cuarta ola" OR variante OR vacuna OR vacunas OR vacunación OR vacunado OR vacunada OR Pfizer OR Moderna OR AstraZeneca OR AZ OR J&J OR Janssen OR "Sputnik V" OR Sinovac OR Novavax OR Curevax OR Covax OR "[Name Minister of Health"] OR @MSPUruguay OR @opsomsuruguay OR OMS OR @WHO) - (is:verified OR RT OR cerveza OR acciones OR fútbol OR partido OR elecciones OR votos OR voto OR votantes OR "nuevos casos" OR "casos confirmados" OR "casos nuevos" OR "casos totales" OR "muertes totales") |
| Canada (FR) | French | profile_country:CA (covid OR covid19 OR covid-19 OR covid_19 OR corona OR coronavirus OR virus OR nCoV OR SARSCoV2 OR infecté OR infectée OR infectées OR contagiuex OR contagion OR pandemie OR "troisieme vague" OR "quatrieme vague" OR variant OR vaccin OR vaccins OR vaccination OR vacciné OR vaccinée OR Pfizer OR Moderna OR AstraZeneca OR AZ OR J&J OR Janssen OR "Sputnik V" OR Sinovac OR Novavax OR Curevax OR Covax OR @GouvCanSante OR OMS OR @WHO) - (is:verified OR RT OR beer OR stocks OR football OR game OR cricket OR elections OR votes OR voters OR vote OR "new cases" OR "total cases" OR "total deaths" OR bière OR bouse OR match OR elections OR votes OR votants OR electeurs OR "nouveaux cas" OR "nombre de décès" OR "nombre de cas") |
| Canada | English, French | profile_country:CA (covid OR covid19 OR covid-19 OR covid_19 OR corona OR coronavirus OR virus OR nCoV OR SARSCoV2 OR infecté OR infectée OR infectées OR contagiuex OR contagion OR pandemie OR "troisieme vague" OR "quatrieme vague" OR variant OR vaccin OR vaccins OR vaccination OR vacciné OR vaccinée OR infection OR infected OR infections OR pandemic OR "3rd wave" OR "4th wave" OR "infection curve"OR vaccine OR jab OR vaccines OR vaccinated OR vaccination OR Pfizer OR Moderna OR AstraZeneca OR AZ OR J&J OR Janssen OR "Sputnik V" OR Sinovac OR Novavax OR Curevax OR Covax OR @GouvCanSante OR @GovCanHealth OR OMS OR @WHO) - (is:verified OR RT OR beer OR stocks OR football OR game OR cricket OR elections OR votes OR voters OR vote OR "new cases" OR "total cases" OR "total deaths" OR bière OR bouse OR match OR elections OR votes OR votants OR electeurs OR "nouveaux cas" OR "nombre de décès" OR "nombre de cas") |
| Nicaragua | Spanish | profile_country:NI (covid OR covid19 OR covid-19 OR covid_19 OR corona OR coronavirus OR virus OR nCoV OR SARSCoV2 OR infección OR infectado OR infectada OR infecciones OR contagio OR contagioso OR contagiado OR contagiada OR contagios OR pandemia OR "tercera ola" OR "cuarta ola" OR variante OR vacuna OR vacunas OR vacunación OR vacunado OR vacunada OR Pfizer OR Moderna OR AstraZeneca OR AZ OR J&J OR Janssen OR "Sputnik V" OR Sinovac OR Novavax OR Curevax OR Covax OR @opsomsnic) - (is:verified OR RT OR cerveza OR acciones OR fútbol OR partido OR elecciones OR votos OR voto OR votantes OR "nuevos casos" OR "casos confirmados" OR "casos nuevos" OR "casos totales" OR "muertes totales") |
| Perú | Spanish | profile_country:PE (covid OR covid19 OR covid-19 OR covid_19 OR corona OR coronavirus OR virus OR nCoV OR SARSCoV2 OR infección OR infectado OR infectada OR infecciones OR contagio OR contagioso OR contagiado OR contagiada OR contagios OR pandemia OR "tercera ola" OR "cuarta ola" OR variante OR vacuna OR vacunas OR vacunación OR vacunado OR vacunada OR Pfizer OR Moderna OR AstraZeneca OR AZ OR J&J OR Janssen OR "Sputnik V" OR Sinovac OR Novavax OR Curevax OR Covax OR @OPSOMSPeru OR @Minsa_Peru) - (is:verified OR RT OR cerveza OR acciones OR fútbol OR partido OR elecciones OR votos OR voto OR votantes OR "nuevos casos" OR "casos confirmados" OR "casos nuevos" OR "casos totales" OR "muertes totales") |
| Trinidad & Tobago | English | profile_country: TT ((covid OR covid19 OR covid-19 OR covid_19 OR corona OR coronavirus OR virus OR nCoV OR SARSCoV2 OR infection OR infected OR infections OR contagion OR contagious OR contagions OR pandemic OR "3rd wave" OR "4th wave" OR "infection curve" OR variant OR vaccine OR jab OR vaccines OR vaccinated OR vaccination OR Pfizer OR Moderna OR AstraZeneca OR AZ OR J&J OR Janssen OR "Sputnik V" OR Sinovac OR Novavax OR Curevax OR Covax OR @MOH_TT) - (is:verified OR RT OR beer OR stocks OR football OR game OR cricket OR elections OR votes OR voters OR vote OR "new cases" OR "total cases" OR "total deaths") |
| Thailand | Thai | profile_country:TH (โควิด OR โควิด-19 OR โควิด_19 OR โคโรนา OR ไวรัสโคโรนา OR ไวรัส OR nCoV OR SARSCoV2 OR การติดเชื้อ OR ติดเชื้อ OR การติดเชื้อ OR การแพร่เชื้อ OR แพร่เชื้อ OR การติดต่อ OR การระบาดใหญ่ OR "ระลอก 3" OR "ระลอก 4" OR กราฟการติดเชื้อ OR สายพันธุ์ OR วัคซีน OR การแทง OR วัคซีน OR ฉีดวัคซีนแล้ว OR การฉีดวัคซีน OR ไฟเซอร์ OR โมเดอร์นา OR แอสตร้าเซนเนก้า OR AZ OR J&J OR แจนเซ่น OR "สปุ๊ตนิค วี" OR ซิโนแวค OR โนวาแวกซ์ OR เคียวร์แวกซ์ OR โคแวกซ์ OR @pr_moph OR @WHOThailand) - (is:verified OR RT OR เบียร์ OR สต็อก OR ฟุตบอล OR เกม OR คริกเก็ต OR การเลือกตั้ง OR การลงคะแนน OR ผู้ลงคะแนน OR ลงคะแนน OR ผู้ติดเชื้อรายใหม่ OR ผู้ติดเชื้อทั้งหมด OR ผู้เสียชีวิตทั้งหมด) |
| Indonesia (Bahasa) | Bahasa | profile_country:ID (Covid OR covid-19 OR covid_19 OR corona OR "virus corona" OR virus OR nCoV OR SARS-CoV-2 OR infeksi OR terinfeksi OR infeksi OR penularan OR menular OR penularan OR pandemi OR "gelombang ke-3" OR "gelombang ke-4" OR "kurva infeksi" OR varian OR vaksin OR suntikan OR vaksin OR divaksinasi OR vaksinasi OR Pfizer OR Moderna OR AstraZeneca OR AZ OR J&J OR Janssen OR "Sputnik V" OR Sinovac OR Novavax OR Curevax OR Covax) - (bir OR pasokan OR "sepak bola" OR permainan OR "permainan kriket" OR OR pemilihan OR suara OR pemilih OR suara OR "kasus baru" OR "jumlah kasus" OR "jumlah kematian") |
| Switzerland | French, Italian, German | profile_country:CH (covid OR covid19 OR covid-19 OR covid_19 OR corona OR coronavirus OR virus OR nCoV OR SARSCoV2 OR Infektion OR infiziert OR Inkektionen OR Ansteckung OR ansteckend OR Ansteckungen OR Pandemie OR "3. Welle" OR "4. Welle" OR Infektionskurve OR Variante OR Impfstoff OR Impfung OR Impfstoffe OR Impfungen OR geimpf OR infecté OR infectée OR infectées OR contagiuex OR contagion OR pandemie OR "troisieme vague" OR "quatrieme vague" OR variant OR vaccin OR vaccins OR vaccination OR vacciné OR vaccinée OR infezione OR infetto OR infezioni OR contagio OR contagiosi OR contagi OR pandemia OR "terza ondata" OR "quarta ondata" OR "curva dei contagi" OR variante OR vaccino OR iniezione OR vaccini OR vaccinato OR vaccinazione OR Pfizer OR Moderna OR AstraZeneca OR AZ OR J&J OR Janssen OR ""Sputnik V"" OR Sinovac OR Novavax OR Curevax OR Covax OR @BAG_OFSP_UFSP OR @WHO) - (is:verified OR RT OR Bier) |
