# Supplementary material for: Using Machine Learning Technology (Early Artificial Intelligence–Supported Response With Social Listening Platform) to Enhance Digital Social Understanding for the COVID-19 Infodemic: Development and Implementation Study
Source: JMIR Infodemiology. 2023 Aug 21;3:e47317. doi: 10.2196/47317 (PMC10477919; doi:10.2196/47317)
Supplement: Multimedia Appendix 4 [file infodemiology_v3i1e47317_app4.docx]

**Multimedia Appendix 4. English and Spanish keywords for each individual category**

**Seedwords for Mexico**

| **Main Categories** | **Sub-categories** | **Seedwords (english)** |
| --- | --- | --- |
| The Cause  How did the virus emerge and how is it spreading? | The Cause of the virus | paciente 0, paciente cero, primer caso, primera infección, originar, originado, derivado de laboratorio, creado en el laboratorio, mercado de Wuhan, mercado humedo, mercados húmedos, mercado de animales, pangolín, pangolines, murciélago, murciélagos, sopa de murciélago, animal salvaje, hecho por el hombre, arma biológica, creado por China, Mercado de Huanan, alimentos congelados importados, radiaciones, virus chino, Virus de China, Chinavirus, Virus CCP, CCPvirus, negacionismo, negacionista, negacionistas, última pandemia, otra pandemia, artificial, fuga de laboratorio, colmillos, en el laboratorio |
|  | Stigma about the spread | racista, prejuicioso, racismo, inmigrantes, virus comunista, migrantes, refugiados, refugiado, xenófobo, xenofobia, aporofobia, antisemita, antisemita, Español, asiáticos, negros, trabajadores domésticos, personas de raza negra, racializado, racializado, musulman, musulmanes, turbante |
|  | Stigma about or by infected people | estigma social, estigmatizado, estigmatizante, estigmatizante, estigma, estigmatizar, sospechoso de covid19, sospechoso de covid-19, sospechoso de covid, sospechoso de corona, sospechoso de coronavirus, cacería de brujas, cacería de brujas, culpa del coronavirus, inmundo, super esparcidor, super esparcidor, deshumanizar, deshumanizar, culpa, responsable de, irresponsable |
| The Illness  What are the symptoms and how is it transmitted ? | Confirmed symptoms | fiebre, tos, sintiendo fatiga, experimentando fatiga, fatigado, sintomático, Anosmia, gusto, oler, pulmón, infección pulmonar, dolor de cabeza, bronquitis, comorbilidad, hiposmia, fuego cerebral, inflamación, neumonía, cansancio, crónico, rinitis, disnea, náusea, vomitando, con síntomas, síntomas claros, tengo síntomas, tiene síntomas, congestión, dolores, escalofríos, no tiene sabor, sin olor, a frio, mental alterado, tiene síntomas, dificultad respiratoria, respiratorio agudo, en aliento |
|  | Other discussed symptoms | garganta, Diarrea, conjuntivitis, migraña, migrañas, mal aliento, erupciones, Dolor de pecho, presión en el pecho, muscular, mialgia, garganta, conjuntivitis, cardiovascular, mal aliento, Dolor de pecho, presión en el pecho, muscular, hipoxia, hipercapnia, enfermedad post-viral, enfermedad post-viral, dolor crónico, disfunción del sueño, Encefalomielitis miálgica, daño duradero, pies de covid, decoloración de los dedos, septicemia, hipoxemia, daño de órganos, estreptococo |
|  | Prolonged Symptoms | covid largo, síntomas prolongados, constante, condición post-covid, covid crónico, crónico, secuela tardía, Covid de largo plazo, covid a largo plazo, posaguda, síntomas tardíos, síntomas persistentes, fatiga cronica |
|  | Modes of transmission | transmitir, transmisión, mucoso, membrana mucosa, fluidos, aéreo, transmisión por aerosol, a través del aire, aerosol, aerotransportado, gotitas respiratorias, gotita, gotas, partículas líquidas, superficie, superficies contaminadas, exposición, fómites, asintomático, asintótico, asymp, sin síntomas, sin síntomas, asintomático, libre de síntomas, presintomático, pre síntomas, pre sintomático, aún por mostrar síntomas, antes de los síntomas, antes de los síntomas, desarrollar síntomas, presintomático, incubación, incubando |
|  | Transmission settings | interior, lugares cerrados, entornos semicerrados, ventilación, filtración de aire, fiesta de Navidad, restaurantes, reunión, Gimnasio, gimnasios, oficinas, bares, discotecas, conciertos, eventos publicos, escuelas, Centros comerciales, prisión, Hotel, Embarcacion, astillero, dormitorios de trabajadores, baños, delirio, aglomeraciones |
|  | Immunity | Re-infección, reinfeccion, reinfectado, inmunidad adquirida, infección natural, anticuerpos, inmunidad, previamente infectado, inmunidad derivada, inmunidad de duración, inmunidad adquirida por infección, previamente expuesto, infectado una vez, dos veces infectado, célula T, células t |
|  | Variants | cepa, mutado, nueva cepa, nueva variante, mutación, variante, alfa, beta, gama, delta, Variante británica, Variante india, Variante de Sudáfrica, Variante sudafricana, Variante brasileña, doble mutación, B.1.1.7, B.1.351, B.1.427, B.1.429 |
|  | Demographic Vulnerability & Risks | ancianos, adultos mayores, vulnerabilidad, vulnerable, grupo en riesgo, grupo de riesgo, grupos de riesgo, viejos, abuelos, adultos mayores, patología, patologias, diabetes, cáncer, diabéticos, asma, hipertensión, Inmunodeprimido, sistema inmunológico comprometido, inmunidad comprometida, abuelo, abuela, anciano, Alta presión sanguínea, obesidad, hiperinflamación, VIH, artritis, dolor artrítico, reumatoide, reumático |
|  | Mental Health | salud mental, salud emocional, crisis de salud mental, enfermedad mental, angustia mental, agotamiento, ansiedad, ansioso, estrés, depresión, post traumático, postraumático, Trastorno de estrés postraumático, trastorno de estrés, frustración, Estoy frustrado, sentirse desanimado, Duelo, dolor, abuso de drogas, insomnio, deprimido, sintiéndome tan deprimido, suicidio, sobredosis , consejería, consejero, doomscrolling, Doomsurf, anorexia, angustia, estado mental, depresión crónica, tristeza crónica, suicida, postraumático |
| The Treatment  How can it be treated or cured ? | Current treatment | tratamiento, tratamientos, antivírico, antibiótico, antibioticos, medicamento, medicamentos, droga, antiinflamatorio, proteasa, dexametasona, esteroides, hormonas, vitamina, vitaminas, vitamina D, vitamina C, medicinas que salva vidas, tratamiento que salva vidas, corticosteroides, oxígeno, recetar, preceptivo |
|  | COVID-19 vaccine | vacuna para el COVID-19, vacuna para el COVID-19, Pfizer, biontech, Moderno, CoronaVac, Sputnik V, BBIBP-CorV, EpiVacCorona, synovac, sputnik, vacuna rusa, Johnson y Johnson, Johnson y Johnson, Johnson y Johnson, covaxina, iqvia, Oxford, astrazeneca, adz1222, Novavax, gsk, curevac, covax, covaxx, vector viral, fosun, sinopharm, canino, adenovirus, plásmido, vida atenuada, subunidad proteica, mRna, BNT162b2, vacuna basada en ARNm, mrna, proteína de pico, material genético, ARN, Calidad de la vacuna, vacuna satisfactoria, efectividad, eficacia, precalificación de vacunas, Fase I, Fase II, fase iii, Aprobación de la FDA, aprobación de la vacuna, EMEA, FDA, vacuna apresurada, mercurio, aluminio, adverso, efectos de la vacuna, eventos adversos, efectos adversos, anafiláctico, anafilaxis, anafilaxia, alérgico, parálisis de Bell, Parálisis de Bell, Síndrome de Guillaume-barre, GBS, TEA, brazo adolorido, respuesta inmune, reacciones severas, a la vacuna, vacuna, inyección de refuerzo, inyección de covid, coágulo de sangre, trombosis, miocarditis, efecto secundario, fatal |
|  | Health care workers (HCW) and vaccine | vacunación de trabajadores de la salud, trabajadores de la salud vacunados, personal de vacunación, personal de vacunas, vacunadores, vacunación de enfermeras, vacunar enfermeras, voluntarios médicos, voluntarios del hospital, vacunador, vacunar a los doctores, Primera línea de EMS, EMT de primera línea, paramédicos de primera línea, voluntarios covid, inoculado, inoculación, trabajador del hospital, EMS, EMT, primeros respondedores, brigadas médicas, trabajador del hospital, primera línea, brigadas médicas, doctores jubilados, brigadas, enfermeras jubiladas |
|  | General vaccine discussion | anti-vacunación, antivacunas, anti-vax, anti-vacuna, contra la vacuna, anti vacuna, anti-vacunas, sin vacuna, vaxxers, las vacunas matan, vacuna contra la polio, vacuna contra el tétanos, vacuna, vacuna contra el virus del papiloma, vacuna contra el VPH, vacuna contra la gripe, vacuna contra la influenza, mmr, vacuna contra el sarampión, varivax, vacuna contra la rubéola, vacuna contra las paperas, vacunas contra la polio, vacunas contra el tétanos, vacunas contra la hepatitis, vacunas contra el virus del papiloma, vacunas contra el VPH, vacunas contra la gripe, vacunas contra la influenza, vacunas contra el sarampión, vacunas contra la rubéola, vacunas contra las paperas, preocupaciones sobre la vacuna, temor a la vacuna, renuencia a la vacuna, Miedo a la vacuna, antivacunas, Indecisos a vacunarse, miedo a las vacunas, Escepticismo sobre las vacunas, turismo de vacunas, turista de vacuna |
|  | Science and R&D | I + D, I y D, investigación y desarrollo, investigar, resultados de la investigación, investigación, investigando, investigador, investigadores, investigador, exploración, resultados de la investigación, recomendaciones, expertos en infecciones, expertos médicos, científicos médicos, revolucionario, descubrimiento, estudio reciente, estudio muestra, estudio mostrado, informacion cientifica, académico, proceso científico, experimentar, experimentando, ensayo clínico, ensayos clínicos, papel cientifico, revisado por pares, revisado por pares, Prueba cientifica, evidencia científica, probado científicamente, lanceta, virólogo, informacion cientifica, ciencia post normal, ciencia posnormal, ciencia |
|  | Non proven treatments | remdesivir, nitazoxanida, inhalar, inhalación, aceite de coco, Coco, radiación, ultravioleta, holístico, homeopatía, homeopático, remedio, remedios, remedios caseros, Vaporización, Vapores, remedio casero, zinc, complejo b, inhalar cocaína, planta artemisa, Lopinavir, enjuague bucal, solución de povidona yodada, Interferón-β1a, anticuerpos monoclonicos, ritonavir, interferón, interferón, azitromicina, meflochin |
|  | Myths | mito, mitos, 5G, beber alcohol, lejía, agua fría, clima frío, Dexametasona, ajo, Secador de manos, aguantar la respiración, clima cálido, Clima húmedo, baño caliente, guindilla, moscas domésticas, hidroxicloroquina, Intoxicación por CO2, metanol, etanol, mosquitos, salina, zapatos limpios, escáner térmico, Lámpara uv, lámpara ultravioleta |
| The Interventions  What is being done by government and health authorities and societal institutions? | Testing | pcr, prueba, antígeno, serológico, cadena de polimerasa, polimerasa, prueba de covid, pruebas de covid, examen covid, exámenes covid, torunda, muestra de hisopo, prueba rápida, hisopo, hisopo largo, kits de prueba, falsos positivos, examen rápido, prueba, prueba de antígenos, seroprevalencia, kary mullis, tomografía computarizada de tórax, RT-PCR, pruebas de inmunodiagnóstico en el lugar de atención, prueba diagnostico rapida, prueba rápida basada en antígenos, análisis de sangre, rRT-PCR, prueba de amplificación de ácido nucleico, NAAT |
|  | Contact Tracing | rastreo de contacto, seguimiento de contactos, rastreo de virus, rastereo de covid, rastro, seguimiento, rastreo, lista de contactos, rastreo de contactos hacia atrás, entrevistas de contacto, seguimiento de contacto directo, contactos directos, contacto directo, vigilancia, trazadores, vigilancia, contacto con, seguimiento y localización, vigilancia, trazadores, caso contacto, en contacto |
|  | Supportive Care | paciente, pacientes, cuidado, enfermeras, enfermero, Personal medico, atención médica de primera línea, UCI, cuidados intensivos, terapia de oxigeno, hidratación, intubación, intubar, médico, cuidado médico, proveedor de cuidado de la salud |
|  | Vaccine distribution and policies on access | distribución de vacunas, Equidad de vacuna, Acceso a vacunas, suministro de vacunas, envío de vacunas' nan 'distribuir, distribución desigual, suministro desigual, logística de vacunas, despliegue de la vacuna, estrategia de vacuna, estrategia de vacunación, escasez de suministro, centro de vacunacion, centros de vacunacion, lugar de vacunación, logística, congelado, ultracongelado, ultracongelado, refrigerado, refrigeración, caja de hielo, cajas de hielo, nacionalismo de vacuna, inmunidad colectiva, inoculación masiva, 1ra inyección, 2da inyección, primera inyección, segunda inyección, 1ra dosis, 2da dosis, primera dosis, segunda dosis, 1er vacuna, Segunda vacuna, primera vacuna, segunda vacuna, dos dosis, tercera dosis |
|  | Personal Measures | auto confinamiento, confinar, confinado, bloqueado, aislar, quedarse en casa, No salir, en casa, usar mascarilla, usando una mascara, cubriendo la boca, máscaras n95, N95, 6 pies, seis pies, distancia, distancia social, distanciamiento social, seis pies de distancia, 2 metros, 1,5 metros, dos metros, Quédate en casa, distanciamiento, preventivo, medidas preventivas, preventivo, gel dispensador, usa la mascarilla, enmascarar, máscarilla puesta |
|  | Measures in public settings | bares cerrados, restaurantes cerrados, reunión de grupo, segregación, separación, protocolo, protocolos, Sin contacto, sin contacto, covid friendly, libre de covid, seguridad, plan de seguridad, publico limitado, bares cerrados, restaurantes cerrados, control de la multitud, pautas, recomendaciones |
|  | Travel Measures | fronteras, frontera, prueba de PCR negativa, formulario de salud, formulario de salud para viaje, 72 horas, durante tu viaje, viajero, viajeros, turistas, viaje, turismo, avión, aviones, aeropuerto, vuelo, vuelos, viaje aéreo, transporte aéreo, viaje en tren, viaje por carretera, vacaciones, día festivo |
|  | Immunity Pass | pasaporte de inmunidad, requisito de entrada, covidpass, pase covid, pase de inmunización, pase de vacuna, pase de salud, prueba de vacunación, pase verde |
|  | Reduction of movement | restricciones, aislamiento, reabrir, toque de queda, mayor conciencia, estado de alarma, bloqueo nacional, bloqueo del perímetro, bloqueo parcial, bloqueo del perímetro, reglas de encierro, nivel, emergencia, estado de emergencia, limitar la movilidad, movilidad |
|  | Protection: medical equipment | profilaxis, EPI, PEP, ropa protectora, gafas de protección, guantes, Lentes, caretas, protectores faciales médicos, vestidos, delantales protectores, protección para los ojos, máscaras quirúrgicas, respiradores |
|  | Health Technology | seguimiento de salud, telemedicina, biometrico, industria 4.0, personalización, Impresión 3d, Impresión 3D, fabricante de salud, tecnología de la salud, automatización, laboratorio de fabricación, telesalud, asesoramiento virtual, ventilador, ventiladores, oxímetro, concentrador de oxígeno |
|  | Digital health technology | geolocalización, cámaras, circuito cerrado de televisión, inteligencia artificial, big tech, computación en la nube, tecnología disponible, wearables, anillo inteligente, anillos inteligentes, reloj inteligente, relojes inteligentes, reloj apple, reloj inteligente, IoT, Internet de las Cosas, aceleración tecnológica, blockchain, aplicaciones móviles, aplicaciones de Android, aplicaciones ios, aplicaciones, relojes inteligentes, reloj inteligente apple, Applewatch, dispositivos inteligentes, robótica |
|  | Pandemic Fatigue | harto, Harto de esto, emocionalmente agotado, fatiga covid, largo covid, constante, a largo plazo, persistir, interminable, sin fin, sin fin, exhausto, agotamiento pandémico, agotamiento por coronavirus, ira pandémica, síntomas duraderos, síndromes persistentes, fatiga cronica, covid eterno |
|  | Faith | Pastor, Predicador, culto, fe, Jehova, Reverendo, evangelista, Misionero, Televangelista, islam, Mezquita, Iglesia, sinagoga, Congregación, protestante, Evangélico, santidad, Biblia, asistentes a la iglesia, oración, Ramadán, Eid al-Fitr, hajj, Peregrinaje, hajj, Hadj, Pascua de Resurrección, Lento, Adviento, mawly, Janucá, Pascua, Pascua, Rosh Hashaná, Yom Kippur, Diwali, año Nuevo Chino, Todos los Santos |
|  | Industry & Economic impact | económico, recesión, pancesión, depresión económica, cierre de negocios, bancarrota, desempleo, desempleado, sin empleo, cerrado, PIB, economía, crisis económica, inflación, mercado negro, mercado informal, sector informal, línea de producción, sector industrial, negocio, pequeña empresa, pequeño, bancarrota, bancario, bancos, nómina de sueldos, comercio, comercio, hiperinflación, gran depresion, bolsa de Valores, extranjero, depresión económica, financiero global, divisa, reactivación, Recuperación económica, Comercio, Sindicato, uniones de la banda de rodadura, futuro del trabajo, teletrabajo, homeoffice |
|  | Environment | ambiental, climático, medio ambiente, tiempo, clima, catástrofe, tornado, huracán, viento, vientos, torrente, lluvias, lluvia, fuego, prender fuego, incendio, ardiendo, deforestación, deforestando, bosque, Arroyo, inundaciones, inundación, sequía, huracán, contaminación, la contaminación del aire, contaminación, inundación, inundación, aguas residuales |
|  | Inequalities & human rights | pobreza, pobre, poder, adinerado, Rico, multimillonario, millonario, multimillonario, altos ingresos, de bajos ingresos, afluente, transferencia de riqueza, inequidad social, empobrecer, Vagabundo, marginación, opresión, privar de derechos civiles, enriquecer, ghetto, desigualdad de género, brecha de género, desigualdad, desigual, ultra rico, seguridad alimentaria, derechos humanos |
|  | Civil Unrest | activista, alboroto, disturbios, violencia publica, malestar público, disturbio publico, desobediencia civil, protesta no violenta, sedición, contramanifestación, contraprotesta, subversión, piquetes, piquete, boicotear, agitadores, huelga salvaje, antifa, disturbios, Huelga, demostración, manifestantes, Touril político, Crisis política, golpe de Estado, golpe, barricada, barricadas, protesta, rebelde, rebeldes, rebelión, revolución |
|  | Youth | guardería, niñera, fiesta de pijamas, pijamada, bar mitzvah, dulces dieciséis, fiesta de piscina, viaje de estudios, viaje de estudios, coronials, juventud, joven, muchacho, jovenes, adolescente' nan 'adolescentes |
| Type of Information  What types of information are most engaging | Statistics & Data | información, Estadísticas, datos, numero de casos, cifras, parámetros, contando, Compilacion, contar, recuento, volumen, número de muertos, números, encuestas, fecha de ubicación, casodemia, modelos matemáticos, modelos de fecha, modelos científicos, estadísticas de coronavirus, estadísticas de covid, estadísticas de covid, graficas, gráficos, comparaciones de gráficos, modelado de datos |
|  | Misinformation & Disinformation | desinformación, no probado, pseudociencia, pseudocientífico, rumor, rumor, rumores, rumores, engañoso, creíble, confiable, de confianza, posverdad, desacreditado, infundado, sin bases, Apocalipsis zombie, apocalipsis, desconfianza, Falta de confianza, desinformación, publicidad, conspiración, clickbait, noticias falsas, manipulación, manipulativo, censura, censurar, conspiración, conspirativo, conspirar, ellos conspiran, conspirar, lecciones, conspirador, desinformación pandémica, infodemia, doble discurso, guerra psicológica, guerra psicológica, guerra psíquica, paranoia |
|  | Sources & Influencers | Fuentes de información, hombre de influencia, influencers, sensibilizar, sensibilización, redes sociales, medios de comunicación social, Tik Tok, twitch, reddit, creador de tendencias, Weixin, wechat, adoptante temprano, disruptivo, revolucinario, medcram, Fuentes de información, Prensa, promedio |

**Seedwords for United Kingdom**

| **Main Categories** | **Sub-categories** | **Seedwords (english)** |
| --- | --- | --- |
| The Cause  How did the virus emerge and how is it spreading? | The Cause of the virus | patient 0, patient zero, first case, first infection, originate, originated, lab derived, created in lab, wuhan market, wet market, wet markets, animal market, pangolin, pangolins, bat, bats, bat soup, wild animal, man made, biological weapon, created by China, Huanan market, imported frozen food, radiations, chinese virus, China virus, Chinavirus, CCP virus, CCPvirus, negationism, negastionist, negationists, last pandemic, another pandemic, manmade, lab leak, fangs, in a lab |
|  | Stigma about the spread | racist, prejudiced, racism, immigrants, communist virus, migrants, refugees, refugee, xenophobic, xenophobia, aporophobia, antisemite, antisemitic, spaniards, asians, blacks, domestic workers, black people, racialised, racialized, muslim, muslims, turban |
|  | Stigma about or by infected people | social stigma, stigmatized, stigmatising, stigmatizing, stigma, stigmatize, covid19 suspect, covid-19 suspect, covid suspect, corona suspect, coronavirus suspect, witch hunt, witchhunt, coronavirus guilt, unclean, superspreader, super-spreader, dehumanize, dehumanise, fault, responsible for, irresponsible |
| The Illness  What are the symptoms and how is it transmitted ? | Confirmed symptoms | fever, cough, feeling fatigue, experiencing fatigue, fatigued, symptomatic, Anosmia, taste, smell, lung, lung infection, headache, bronchitus, comorbidity, hyposmia, brain fog , inflammation, pneumonia, tiredness, chronical, coryza, dyspnoea, nausea, vomiting, with symptoms, clear symptoms, i have symptoms, has symptoms, congestion, aches, chills, no taste, no smell, a cold, altered mental, have symptoms, respiratory distress, acute respiratory, no breath |
|  | Other discussed symptoms | throat, diarrhea, conjunctivitis, migraine, migraines, bad breath, rashes, chest pain, chest pressure, muscular, Myalgia, throat, conjunctivitis, cardiovascular, bad breath, chest pain, chest pressure, muscular, hypoxia, hypercapnia, post-viral disease, post-viral disease, chronic pain , sleep dysfunction, Myalgic Encephalomyelitis, lasting damage, Covid toes , discolouration of fingers , Sepsis, hypoxaemia, organ damage, strep |
|  | Prolonged Symptoms | long covid, prolonged symptoms, lingering, post-covid condition, chronic covid, chronic, late sequelae, long haul covid, long-term covid, post-acute, late symptoms, persistent symptoms, chronic fatigue |
|  | Modes of transmission | transmit, transmission, mucous, mucous membrane, fluids, aerial, aerosol transmission, through the air, aerosol, airborne, respiratory droplets, goticular, droplets, liquid particles, surface, contaminated surfaces , exposure, fomites, asymptomatic, asymptotic, asymp, no symptoms, without symptoms, symptomless, symptom free, presymptomatic, pre symptoms, pre symptomatic, yet to show symptoms, before symptoms, prior to symptoms, developing symptoms, pre-symptomatic, incubation, incubating |
|  | Transmission settings | indoor, closed settiings, semi-closed settings, ventilation, air filtration, christmas party, restaurants, gathering, gym, gymnasiums, offices, bars, nightclubs, concerts, public events, Schools, Shopping malls, Prison, Hotel, Ship, Shipyard, Worker dormitories, restrooms, rave, agglomerations |
|  | Immunity | Re-infection, reinfection, reinfected, acquired immunity, natural infection, antibodies, immunity, previously infected, infection-derived immunity, immunity duration, infection-acquired immunity, previously exposed, infected once, infected twice, t-cell, t-cells |
|  | Variants | strain, mutated, new strain, new variant, mutation, variant, alpha, beta, gamma, delta, British variant, Indian variant, South Africa variant, South African variant, Brazilian variant, double mutation, B.1.1.7, B.1.351, B.1.427, B.1.429 |
|  | Demographic Vulnerability & Risks | seniors, senior citizens, vulnerability, vulnerable, at-risk group, risk group, risk groups, old people, grandparents, older adults, pathology, pathologies, diabetes, cancer, diabetics, asthma, hypertension, Immunocompromised, compromised immune system, compromised immunity, grandfather, grandmother, elderly , high blood pressure, obesity, hyperinflammation, HIV, arthritis, arthritic pain, rheumatoid, rheumatic |
|  | Mental Health | mental health, emotional health, mental health crisis, mental illness, mental distress, burnout, anxiety, anxious, stress, depression, post traumatic, posttraumatic, PTSD, stress disorder, frustration' "i'm frustrated" 'feeling low, Bereavement, grief, drug abuse, insomnia, feeling down, feeling so down, suicide, drug overdose, councelling, councellor, doomscrolling, doomsurfing, anorexia' nan nan nan 'anguish, mental state, chronic depresison, chronic sadness, suicidal, posttraumatic |
| The Treatment  How can it be treated or cured ? | Current treatment | treatment, treatments, antiviral, antibiotic, antibiotics, medicine, medicines, drug, anti-inflammatory, protease, dexamethasone, steroids, hormones, vitamin, vitamins, vitamin d, vitamin c, life saving drug, life saving treatment, corticosteroids, oxygen, prescribe, prescriptive |
|  | COVID-19 vaccine | covid vaccine, covid-19 vaccine, pfizer, biontech, moderna, CoronaVac, Sputnik V, BBIBP-CorV, EpiVacCorona, sinovac, sputnik, russian vaccine, johnson&johnson, johnson & johnson, johnson and johnson, covaxin, iqvia, oxford, astrazeneca, adz1222, novavax, gsk, curevac, covax, covaxx, viral vector, fosun, sinopharm, cansino, adenovirus, plasmid, live-attenuated, protein subunit, mRna, BNT162b2, mRNA-based vaccine, mrna, spike protein, genetic material, RNA, Vaccine quality, vaccine satefy, effectiveness, efficacy, vaccine prequalification, phase i, phase ii, phase iii, fda approval, vaccine approval, EMEA, FDA, rushed vaccine, mercury, aluminum, adverse, vaccine effects, adverse events, adverse effects, anaphylactic, anaphylaxia, anaphylaxis, allergic reaction' "bell's palsy" 'bell palsy, Guillaume-barre syndrome, GBS, ASD, sore arm, immune response, grave reactions, reaction to the vaccine, vaccine reaction, booster shot, covid shot, blood clot, thrombosis, myocarditis, side effect, fatal reaction |
|  | Health care workers (HCW) and vaccine | hcw vaccination, hcw vaccinated, vaccination staff, vaccine staff, vaccinators, nurses vaccinating, vaccinating nurses, medical volunteers, hospital volunteers, vaccinator, vaccinating doctors, frontline EMS, frontline EMTs, frontline paramedics, covid volunteers, innoculated, innoculation, hospital worker, EMS, EMT, first responders, medical brigades, hospital worker, first responders, medical brigades, retired doctors, brigades, retired nurses |
|  | General vaccine discussion | anti-vaccination, anti-vaxxers, anti-vax, anti-vaccine, against vaccine, anti vaccine, antivaccine, no vaccine, vaxxers, vaccines kill, polio vaccine, tetanus vaccine, hepatitis vaccine, papillomavirus vaccine, hpv vaccine, flu vaccine, influenza vaccine, mmr, measles vaccine, varivax, rubella vaccine, mumps vaccine, polio vaccines, tetanus vaccines, hepatitis vaccines, papillomavirus vaccines, hpv vaccines, flu vaccines, influenza vaccines, measles vaccines, rubella vaccines, mumps vaccines, vaccine concerns, vaccine-injury, vaccine hesitancy , vaccine hesitant, antivaccine, vaccine hesitant, vaccine fear, Vaccine scepticism, vaccine tourism, vaccine tourist |
|  | Science and R&D | R&D, R & D, research and development, research, research findings, investigation, doing research, investigator, researchers, researcher, exploration, research findings, findings, infection experts, medical experts, medical scientists, groundbreaking, breakthrough, recent study, study shows, study showed, scientific information, academic, scientific process, experiment, experimenting, clinical trial, clinical trials, scientific paper, peer reviewed, peer-reviewed, scientific proof, scientific evidence, scientifically proven, the lancet, virologist, scientific information, post-normal science, postnormal science, science |
|  | Non proven treatments | remdesivir, nitazoxanide, inhale, inalation, coconut oil, coconut, radiation, ultraviolet, holistic, homeopathy, homeopathic, remedy, remedies, home remedies, vapor level, vapour level, home remedy, zinc, b-complex, snort cocaine, artemisia plant, Lopinavir, mouth wash, povidone iodine solution, Interferon-β1a, monoclonal antibodies, ritonavir, interferon, interferon, azithromycin, mefloquine |
|  | Myths | myth, myths, 5G, drink alcohol, bleach, cold water, cold weather, Dexamethasone, garlic, hand dryer, holding breath, hot climate, humid clinate, hot bath, hot pepper, houseflies, hydroxychloroquin, CO2 intoxication, methanol, ethanol, mosquitoes, saline, clean shoes, thermal scanner, UV lamp, ultra-violet lamp |
| The Interventions  What is being done by government and health authorities and societal institutions? | Testing | pcr, test, antigen, serological, polymerase chain, polymerase, covid test, covid tests, covid exam, covid exams, swab, swab sample, rapid testing, q-tip, long q-tip, testing kits, false positives, quick test, antibodies test, antigens test, seroprevalence, kary mullis, chest computed tomography, RT‐PCR, point-of-care immunodiagnostic tests, rapid diagnostic test, rapid antigen based test, blood tests, rRT-PCR, nucleic acid amplification testing , NAATs |
|  | Contact Tracing | contact trace, contact tracing, virus tracking, covid tracking, trace, tracking, tracing, contact listing, backwards contact tracing, contact interviews, forward contact tracing, direct contacts, direct contact, pharmacovigilance, tracers, monitoring, contact with , track-and-trace, pharmacovigilance, tracers, was in contact, in contact |
|  | Supportive Care | patient, patients, care, nurses, nurse, medical staff, frontline healthcare, icu, intensive care, oxygen therapy, hydration, intubation, intubate, physician, physician care, healthcare provider |
|  | Vaccine distribution and policies on access | Vaccine distribution, Vaccine equity, Vaccine access, vaccine supply, vaccine shipment' nan 'distribution, unequal distribution, unequal supply, vaccine logistics, vaccine rollout, vaccine strategy, vaccionation strategy, supply shortages, vaccination centre, vaccination centres, vaccination hub, logistics, frozen, ultrafrozen, ultra-frozen, refrigerated, refrigeration, ice box, ice boxes, vaccine nationalism, herd immunity, mass innoculation, 1st jab, 2nd jab, first jab, second jab, 1st dose, 2nd dose, first dose, second dose, 1st shot, 2nd shot, first shot, second shot, two doses, third dose |
|  | Personal Measures | self-confinement, confine, confined, locked up, isolate, staying home, no go out, at home, wear a mask, wearing a mask, covering mouth, n95 masks, n95, 6 feet, six feet, distance, social distance, social distancing, six feet apart, 2 meters, 1,5 meters, two meters, stay at home, distancing, preventive, preventive measures, preventative, gel dispenser, use a mask, mask up, mask on |
|  | Measures in public settings | bars closed, restaurants closed, group meeting, segregation, separation, protocol, protocols, no contact, contactless, covid-friendly, covid-free, safety, safety plan, limited public, closed bars, closed restaurants, crowd control, guidelines, recommendations |
|  | Travel Measures | borders, border, negative PCR test, health form , travel health form, 72 hours, during your trip, traveller, travellers, tourists, travel, tourism, airplane, airplanes, airport, aeroplane, aeroplanes, air travel, air transport, train travel, road travel, vacation, holiday |
|  | Immunity Pass | immunity passport, entry requirement, covidpass, covid pass, immunisation pass, vaccine pass, health pass, vaccination proof, green pass |
|  | Reduction of movement | restrictions, lockdown, reopen, curfew, heightened awareness, state of alarm, national lockdown, perimeter lockdown, partial lockdown, perimetral lockdown, lockdown rules, tier, emergency, state of emergency, limit mobility, mobility |
|  | Protection: medical equipment | prophylaxis, PPE, PPEs, protective clothes, goggles, gloves, glasses, face shields, medical face shields, gowns, aprons, eye protection, surgical masks, respirators |
|  | Health Technology | health tracking, telemedicine, biometric, industry 4.0, personalization, 3D printing, 3D print, health maker, health tech, automation, fab lab, telehealth, virtual counseling, ventilator, ventilators, oximeter, oxygen concentrator |
|  | Digital health technology | geolocation, cameras, cctv, artificial intelligence, big tech, cloud computing, wereable technology, wearables, oura ring, oura rings, smart watch, smart watches, apple watch, smartwatch, IoT, Internet of things, techceleration, blockchain, mobile apps, android apps, ios apps, apps, smartwatches, Apple smartwatch, Applewatch, smart devices, robotics |
|  | Pandemic Fatigue | fed up, sick of this, emotionally drained, covid fatigue, long covid , lingering, long-term, linger, endless, never-ending, neverending, exhausted, pandemic burnout, coronavirus burnout, pandemic anger, lasting symptoms, persistent syndroms, chronic fatigue, eternal covid |
|  | Faith | Pastor, Preacher, cult, faith, Jehova, Reverend, Evangelist, Missionary , Televangelist, Islam , Mosque, Church , Synagogue , Congregation , Protestant, Evangelical, Holiness , Bible, church-goers, prayer, Ramadan, Eid al-Fitr, Hajj, Pilgrimage, Haj, Hadj, Easter, Lent, Advent, Mawlid, Hanukkah, Passover, Pesach, Rosh Hashanah, Yom Kippur, Diwali, Chinese New Year, All Saints Day |
|  | Industry & Economic impact | economic, recession, pancession, economic depression, businesses closing, bankruptcy, unemployment, jobless, joblessness, shuttered, GDP, economy, economic crisis, inflation, black market, informal market, informal sector, production line, industrial sector, Business, small company, small companies, bankruptcy, banking, banks, payroll, trade, trading, hyperinflation, great depression, stock market, foreign investment, economic depression, global financial, currency, reactivation, Economic recovery, Commerce, trade union, treade unions, future of work, teleworking, home office |
|  | Environment | environmental, climatic, environment, weather, climate, catastrophe, twister, hurricane, wind, winds, torrential, rains, rain, fire, set fire, burning, ablaze, deforestation, deforesting, deforest, stream, floods, flood, drought, hurricane, pollution, air pollution, contamination, flooding, inundation, wastewater |
|  | Inequalities & human rights | poverty, poor, wealth, wealthy , rich, billionaire, millionaire, multimillionaire, high income, low income , affluent, wealth transfer, social inequity, impoverish, homeless, marginalization, oppression, disenfranchise, enrich, ghetto, gender inequality, gender gap, inequality, unequal, ultrarich, food insecurity, human rights, ' |
|  | Civil Unrest | activist, riot, rioting, public violence, public unrest, public disturbance, civil disobedience, nonviolent protest, sedition, counterdemonstration, counter protest, subversion, picketing, picket line, boycott, agitators, wildcat strike, antifa, riots, strike, demonstration, demonstrators, political turnoil, political crisis' "coup d'etat" 'coup, barricade, barricades, protest, rebel, rebels, rebellion, revolution |
|  | Youth | babysitting, babysitter, sleepover, slumber party , bar mitzvah, sweet sixteen, pool party , field trip, school trip , coronials, youth, young, youngster, youngsters, teen, teenarger, teens, teenagers |
| Type of Information  What types of information are most engaging | Statistics & Data | information, statistics, stats, number of cases, figures, parameters, counting, compilation, recount, recounting, volume , body count, numbers, surveys, location data, casedemic, mathematical models, data models, scientific models, coronavirus statistics, covid stats, covid statistics, graphs, charts, graph comparisons, data modeling |
|  | Misinformation & Disinformation | misinformation, unproved, pseudoscience, pseudoscientific, rumor, rumour, rumors, rumours, misleading, credible, trustworthy, trusted, post-truth, debunked, baseless, unfounded, zombie apocalypse, apocalypse, distrust, lack of trust, disinformation, propaganda, conspiracy, clickbait, fake news, manipulation, manipulative, censorship, censor, conspiracy, conspirative, conspire, they conspire, conspires, lies, conspiratorial, disinformation pandemic, infodemic, doublespeak, psych warfare, psychological warfare, psywar, paranoia |
|  | Sources & Influencers | Sources of information, influencer, influencers, sensitize, sensitization, social networks, social media, tiktok, twitch, reddit, trendsetter, weixin, wechat, early adopter, gamechanger, game changer, medcram, Sources of information, press, media |
