# Supplementary material for: Using Machine Learning Technology (Early Artificial Intelligence–Supported Response With Social Listening Platform) to Enhance Digital Social Understanding for the COVID-19 Infodemic: Development and Implementation Study
Source: JMIR Infodemiology. 2023 Aug 21;3:e47317. doi: 10.2196/47317 (PMC10477919; doi:10.2196/47317)
Supplement: Multimedia Appendix 5 [file infodemiology_v3i1e47317_app5.docx]

**Multimedia Appendix 5. Precision and recall metrics for algorithm vs boolean methods for all categories**

| Main Categories | Sub-categories | Spanish Algorithm – Precision % | Spanish Boolean – Precision % | English Algorithm – Recall % | English Boolean – Recall % |
| --- | --- | --- | --- | --- | --- |
| The Cause  How did the virus emerge and how is it spreading? | The Cause of the virus | 49.69 | 43.24 | 74.70 | 52.63 |
|  | Stigma about the spread | 29.75 | 26.66 | 54.54 | 52.98 |
|  | Stigma about or by infected people | 66.26 | 54.73 | 47.76 | 44.45 |
| The Illness  What are the symptoms and how is it transmitted ? | Confirmed symptoms | 48.63 | 51.68 | 52.87 | 50.24 |
|  | Other discussed symptoms | 12.17 | 10.82 | 20.69 | 21.43 |
|  | Prolonged Symptoms | 57.38 | 8.01 | 96.15 | 87.01 |
|  | Modes of transmission | 41.54 | 8.33 | 87.78 | 38.28 |
|  | Transmission settings | 51.89 | 28.38 | 62.60 | 55.03 |
|  | Immunity | 86.27 | 84.44 | 91.67 | 93.34 |
|  | Variants | 80.89 | 76.98 | 76.54 | 59.24 |
|  | Demographic Vulnerability & Risks | 61.37 | 52.49 | 59.49 | 44.44 |
|  | Mental Health | 38.15 | 25.09 | 87.76 | 72.12 |
| The Treatment  How can it be treated or cured ? | Current treatment | 1.9 | 2.7 | 72.61 | 45.36 |
|  | COVID-19 vaccine | 32.33 | 38.21 | 43.00 | 35.29 |
|  | Health care workers (HCW) and vaccine | 5.82 | 1.29 | 56.75 | 31.75 |
|  | General vaccine discussion | 42.23 | 27.21 | 65.51 | 51.99 |
|  | Science and R&D | 28.09 | 23.21 | 69.09 | 55.27 |
|  | Non proven treatments | 53.69 | 55.04 | 80.00 | 78.16 |
|  | Myths | 41.86 | 43.24 | 46.33 | 41.15 |
| The Interventions  What is being done by government and health authorities and societal institutions? | Testing | 80.22 | 55.64 | 88.89 | 60.58 |
|  | Contact Tracing | 56.73 | 55.17 | 80.73 | 76.57 |
|  | Supportive Care | 74.12 | 51.67 | 53.79 | 20.91 |
|  | Vaccine distribution and policies on access | 26.47 | 18.91 | 75.61 | 52.09 |
|  | Personal Measures | 53.60 | 48.68 | 76.85 | 53.51 |
|  | Measures in public settings | 34.62 | 35.99 | 79.79 | 64.00 |
|  | Travel Measures | 69.41 | 58.16 | 75.30 | 59.53 |
|  | Immunity Pass | 53.30 | 45.46 | 73.90 | 60.95 |
|  | Reduction of movement | 37.03 | 25.11 | 78.61 | 54.77 |
|  | Protection: medical equipment | 65.79 | 67.12 | 87.37 | 84.16 |
|  | Health Technology | 58.17 | 57.56 | 73.41 | 68.75 |
|  | Digital health technology | 64.90 | 62.74 | 68.75 | 43.14 |
|  | Pandemic Fatigue | 38.99 | 46.15 | 58.90 | 56.61 |
|  | Faith | 55.78 | 50.35 | 67.46 | 60.87 |
|  | Industry & Economic impact | 48.60 | 41.13 | 69.35 | 53.06 |
|  | Environment | 63.94 | 56.97 | 86.17 | 78.44 |
|  | Inequalities & human rights | 58.48 | 54.55 | 71.69 | 58.46 |
|  | Civil Unrest | 41.13 | 34.49 | 66.67 | 66.67 |
|  | Youth | 82.66 | 44.86 | 89.50 | 71.49 |
| Type of Information  What types of information are most engaging | Statistics & Data | 15.61 | 13.27 | 77.05 | 54.10 |
|  | Misinformation & Disinformation | 57.99 | 53.54 | 85.57 | 75.54 |
|  | Sources & Influencers | 60.81 | 53.90 | 90.72 | 74.57 |
