# Supplementary material for: Using Machine Learning Technology (Early Artificial Intelligence–Supported Response With Social Listening Platform) to Enhance Digital Social Understanding for the COVID-19 Infodemic: Development and Implementation Study
Source: JMIR Infodemiology. 2023 Aug 21;3:e47317. doi: 10.2196/47317 (PMC10477919; doi:10.2196/47317)
Supplement: Multimedia Appendix 6 [file infodemiology_v3i1e47317_app6.docx]

**Multimedia Appendix 6. Number of velocities alerts by category from Dec 2020 - Feb 2022**

|  | Mexico | | | United Kingdom | | |
| --- | --- | --- | --- | --- | --- | --- |
| Categories | Total n of Velocity Alerts | Velocity alerts by gender | | Total n of Velocity Alerts | Velocity alerts by gender | |
|  |  | Men | Women |  | Men | Women |
| The cause of the virus | 31 | 15 | 16 | 27 | 13 | 14 |
| Stigma around the spread | 33 | 18 | 15 | 26 | 13 | 13 |
| Stigma about or by infected people | 32 | 16 | 16 | 28 | 14 | 14 |
| Confirmed Symptoms | 28 | 15 | 13 | 24 | 11 | 13 |
| Other discussed symptoms | 29 | 14 | 15 | 22 | 12 | 10 |
| Prolonged symptoms | 23 | 14 | 9 | 27 | 14 | 13 |
| Modes of transmission | 37 | 16 | 21 | 35 | 17 | 18 |
| Transmission settings | 31 | 17 | 14 | 33 | 18 | 15 |
| Immunity | 26 | 18 | 18 | 40 | 21 | 19 |
| COVID-19 Variants | 33 | 17 | 16 | 31 | 16 | 15 |
| Demographic vulnerability & risks | 29 | 14 | 15 | 27 | 14 | 13 |
| Impact on mental health | 29 | 14 | 15 | 24 | 14 | 10 |
| Current treatments | 31 | 14 | 17 | 22 | 13 | 9 |
| COVID 19 Vaccine | 31 | 15 | 16 | 29 | 16 | 13 |
| Health Care Workers & Vaccine | 31 | 16 | 15 | 32 | 18 | 14 |
| General Vaccine Discussion | 38 | 19 | 19 | 30 | 15 | 15 |
| Science and R&D | 33 | 17 | 16 | 28 | 15 | 13 |
| Non-proven treatments | 29 | 15 | 14 | 24 | 12 | 12 |
| Myths | 29 | 15 | 14 | 19 | 9 | 10 |
| Testing | 27 | 12 | 15 | 25 | 13 | 12 |
| Contact tracing | 32 | 16 | 16 | 31 | 17 | 14 |
| Supportive care | 26 | 12 | 14 | 24 | 13 | 11 |
| Vaccine distribution and policies on access | 28 | 13 | 15 | 25 | 12 | 13 |
| Personal measures | 24 | 14 | 10 | 25 | 13 | 12 |
| Measures in public settings | 29 | 16 | 13 | 17 | 10 | 7 |
| Travel measures | 27 | 14 | 13 | 22 | 10 | 12 |
| Immunity pass | 7 | 5 | 2 | 23 | 11 | 12 |
| Reduction of domestic movement | 27 | 14 | 13 | 25 | 14 | 11 |
| Protection: medical equipment | 30 | 16 | 14 | 31 | 14 | 17 |
| Health Technology | 26 | 12 | 14 | 26 | 12 | 14 |
| Digital Health Technology | 30 | 13 | 17 | 20 | 11 | 9 |
| Pandemic fatigue | 31 | 18 | 13 | 24 | 13 | 11 |
| Faith | 24 | 12 | 12 | 28 | 13 | 15 |
| Industry & economic impact | 23 | 12 | 11 | 24 | 11 | 13 |
| Environment | 26 | 13 | 13 | 25 | 12 | 13 |
| Inequalities & Human Rights | 28 | 15 | 13 | 23 | 12 | 11 |
| Civil Unrest | 28 | 14 | 14 | 34 | 16 | 18 |
| Youth | 39 | 19 | 20 | 30 | 16 | 14 |
| Statistics & Data | 33 | 19 | 14 | 30 | 15 | 15 |
| Mis- and disinformation | 31 | 14 | 17 | 25 | 12 | 13 |
| Sources & Influencers | 27 | 13 | 14 | 28 | 13 | 15 |
