# Supplementary material for: Using Machine Learning Technology (Early Artificial Intelligence–Supported Response With Social Listening Platform) to Enhance Digital Social Understanding for the COVID-19 Infodemic: Development and Implementation Study
Source: JMIR Infodemiology. 2023 Aug 21;3:e47317. doi: 10.2196/47317 (PMC10477919; doi:10.2196/47317)
Supplement: Multimedia Appendix 7 [file infodemiology_v3i1e47317_app7.docx]

**Multimedia Appendix 7. Number of velocities alerts by category and question filter from Dec 2020 - Feb 2022**

|  | Mexico | | | United Kingdom | | |
| --- | --- | --- | --- | --- | --- | --- |
| Categories | Total n of Velocity Alerts in questions | Velocity alerts by gender - questions | | Total n of Velocity Alerts in questions | Velocity alerts by gender - questions | |
|  |  | Men | Women |  | Men | Women |
| The cause of the virus | 34 | 17 | 17 | 29 | 13 | 16 |
| Stigma around the spread | 32 | 16 | 16 | 30 | 12 | 18 |
| Stigma about or by infected people | 32 | 14 | 18 | 25 | 13 | 12 |
| Confirmed Symptoms | 37 | 19 | 18 | 19 | 10 | 9 |
| Other discussed symptoms | 45 | 25 | 20 | 35 | 18 | 17 |
| Prolonged symptoms | 35 | 21 | 14 | 22 | 10 | 12 |
| Modes of transmission | 40 | 19 | 21 | 20 | 8 | 12 |
| Transmission settings | 27 | 12 | 15 | 18 | 9 | 9 |
| Immunity | 39 | 22 | 17 | 20 | 9 | 11 |
| COVID-19 Variants | 34 | 17 | 17 | 19 | 10 | 9 |
| Demographic vulnerability & risks | 24 | 11 | 12 | 14 | 7 | 7 |
| Impact on mental health | 32 | 15 | 17 | 24 | 13 | 11 |
| Current treatments | 26 | 10 | 16 | 24 | 10 | 14 |
| COVID 19 Vaccine | 26 | 12 | 14 | 10 | 4 | 6 |
| Health Care Workers & Vaccine | 18 | 7 | 11 | 35 | 16 | 19 |
| General Vaccine Discussion | 39 | 23 | 16 | 23 | 10 | 13 |
| Science and R&D | 38 | 19 | 19 | 14 | 5 | 9 |
| Non-proven treatments | 35 | 16 | 19 | 34 | 17 | 17 |
| Myths | 42 | 20 | 22 | 25 | 10 | 15 |
| Testing | 27 | 11 | 16 | 23 | 11 | 12 |
| Contact tracing | 38 | 18 | 20 | 21 | 9 | 12 |
| Supportive care | 38 | 17 | 21 | 18 | 9 | 9 |
| Vaccine distribution and policies on access | 42 | 21 | 21 | 21 | 8 | 13 |
| Personal measures | 28 | 12 | 16 | 17 | 11 | 6 |
| Measures in public settings | 42 | 21 | 21 | 22 | 10 | 12 |
| Travel measures | 28 | 13 | 15 | 12 | 5 | 7 |
| Immunity pass | 0 | 0 | 0 | 21 | 11 | 10 |
| Reduction of domestic movement | 29 | 13 | 16 | 28 | 13 | 5 |
| Protection: medical equipment | 39 | 20 | 19 | 29 | 12 | 17 |
| Health Technology | 39 | 19 | 20 | 40 | 19 | 21 |
| Digital Health Technology | 40 | 19 | 21 | 39 | 21 | 18 |
| Pandemic fatigue | 30 | 20 | 10 | 34 | 18 | 16 |
| Faith | 40 | 20 | 20 | 25 | 8 | 17 |
| Industry & economic impact | 33 | 14 | 19 | 20 | 12 | 8 |
| Environment | 38 | 18 | 20 | 22 | 9 | 13 |
| Inequalities & Human Rights | 32 | 15 | 17 | 20 | 11 | 9 |
| Civil Unrest | 32 | 16 | 16 | 30 | 12 | 18 |
| Youth | 31 | 16 | 15 | 22 | 9 | 13 |
| Statistics & Data | 39 | 20 | 19 | 15 | 7 | 8 |
| Mis- and disinformation | 36 | 17 | 19 | 13 | 5 | 8 |
| Sources & Influencers | 29 | 12 | 17 | 19 | 10 | 9 |
